# Flow cytometry-based FRET identifies binding intensities in PPARγ1 protein-protein interactions in living cells

**DOI:** 10.7150/thno.29367

**Published:** 2019-07-28

**Authors:** Verena Trümper, Andreas von Knethen, Annegret Preuß, Eugeny Ermilov, Steffen Hackbarth, Laura Kuchler, Sandra Gunne, Anne Schäfer, Tobias Bornhütter, György Vereb, Lázló Ujlaky-Nagy, Bernhard Brüne, Beate Röder, Michael Schindler, Michael J. Parnham, Tilo Knape

**Affiliations:** 1 Institute of Biochemistry I - Pathobiochemistry, Faculty of Medicine, Goethe University Frankfurt, Theodor-Stern-Kai 7, 60590 Frankfurt/Main, Germany; 2 Branch for Translational Medicine and Pharmacology, Fraunhofer Institute for Molecular Biology and Applied Ecology IME, Theodor-Stern-Kai 7, 60596 Frankfurt/Main, Germany; 3 Department of Physics, Humboldt University Berlin, Newtonstraße 15, 12489 Berlin, Germany; 4 Department of Biophysics and Cell Biology, Faculty of Medicine, University of Debrecen, Egyetem tér 1, 4032 Debrecen, Hungary; 5 MTA-DE Cell Biology and Signaling Research Group, Faculty of Medicine, University of Debrecen, Egyetem tér 1, 4032 Debrecen, Hungary; 6 Institute of Medical Virology and Epidemiology of Viral Diseases, University Hospital Tübingen, Karls University Tübingen, Elfriede-Aulhorn-Str. 6, 72076 Tübingen

**Keywords:** binding affinity and intensity, co-localization analysis, flow cytometry-based FRET assay, FRET, N-CoR2, NHR co-factors protein-protein interactions, PPARγ1, RXRα

## Abstract

PPARγ is a pharmacological target in inflammatory and metabolic diseases. Upon agonistic treatment or following antagonism, binding of co-factors is altered, which consequently affects PPARγ-dependent transactivation as well as its DNA-independent properties. Therefore, establishing techniques to characterize these interactions is an important issue in living cells.

**Methods:** Using the FRET pair Clover/mRuby2, we set up a flow cytometry-based FRET assay by analyzing PPARγ1 binding to its heterodimerization partner RXRα. Analyses of PPARγ-reporter and co-localization studies by laser-scanning microscopy validated this system. Refining the system, we created a new readout to distinguish strong from weak interactions, focusing on PPARγ-binding to the co-repressor N-CoR2.

**Results:** We observed high FRET in cells expressing Clover-PPARγ1 and mRuby2-RXRα, but no FRET when cells express a mRuby2-RXRα deletion mutant, lacking the PPARγ interaction domain. Focusing on the co-repressor N-CoR2, we identified in HEK293T cells the new splice variant N-CoR2-ΔID1-exon. Overexpressing this isoform tagged with mRuby2, revealed no binding to Clover-PPARγ1, nor in murine J774A.1 macrophages. In HEK293T cells, binding was even lower in comparison to N-CoR2 constructs in which domains established to mediate interaction with PPARγ binding are deleted. These data suggest a possible role of N-CoR2-ΔID1-exon as a dominant negative variant. Because binding to N-CoR2-mRuby2 was not altered following activation or antagonism of Clover-PPARγ1, we determined the effect of pharmacological treatment on FRET intensity. Therefore, we calculated flow cytometry-based FRET efficiencies based on our flow cytometry data. As with PPARγ antagonism, PPARγ agonist treatment did not prevent binding of N-CoR2.

**Conclusion:** Our system allows the close determination of protein-protein interactions with a special focus on binding intensity, allowing this system to characterize the role of protein domains as well as the effect of pharmacological agents on protein-protein interactions.

## Introduction

Förster´s resonance energy transfer (FRET) between fluorescent proteins is an elegant non-invasive technique available to detect direct protein-protein interactions in living cells. It is based upon the energy transfer from an excited donor fluorophore to an adjacent acceptor fluorophore, resulting in decreased fluorescence emission by the donor and enhanced fluorescence emission by the acceptor 1-3. For direct protein-protein interactions, FRET is highly dependent on the distance between the two fluorophores. Accordingly, this phenomenon only occurs when the distance between donor and acceptor ranges between 1-10 nm and the emission spectra of the donor overlaps with the excitation spectra of the acceptor 4. In addition, FRET is also dependent on the geometry of the fluorophores in a donor-acceptor FRET pair.

A large number of FRET methods, such as fluorescence lifetime imaging microscopy (FLIM), time-correlated single photon counting (TCSPC) or time-resolved (TR) energy transfer by TR-FRET, have been developed to measure fluorescence lifetime over the last few years. However, major limitations still exist 5-7. Mostly, FRET measurements are done by complex fluorescence microscopy or analysis, which allows only the analysis of a small number of cells and essentially precludes high-throughput-screening (HTS) for protein interactions.

A new innovative method to overcome these limitations is to detect and quantify FRET by flow cytometry 8, 9. This non-invasive, sensitive and quantitative method allows the study of direct protein-protein interactions in large numbers of living cells and samples in a reasonable amount of time. Another new FRET-based method is related to the assessment of the binding affinity based on the determination of the FRET intensity via the measurement of the median fluorescence intensities (MFI) value to calculate the flow cytometry-based FRET efficiencies to rate the binding strength and thus, it allows detection of changes in the affinity and intensity of protein-protein interactions 10-13.

Cyan and yellow fluorescent proteins (CFPs and YFPs) are generally used as FRET fluorophores. However, photophysical aspects of CFPs and YFPs, like rapid multi-rate and reversible photobleaching are problematic for FRET 14. Consequently, many CFP- and YFP-based FRET reporters produce only small changes in FRET. Alternative FRET pairings between a green fluorescent protein (GFP) or YFP and an orange or red fluorescent protein (OFP or RFP) have been explored, but still have major limitations, i.e. low signal-to-noise ratio. Consequently, FRET efficiency and shifts in terms of fluorescence intensity upon FRET are too small to measure subtle differences in alterations of protein binding, for instance disturbance of interaction by drugs. A systematically developed, new FRET pair to overcome and minimize these limitations is based on Clover/mRuby2, one of the brightest GFP-based, respectively, brightest RFP-based fluorescence probes characterized so far, with properties better suited to a wide range of FRET applications 14.

In recent years, a few new drug screening systems have been developed to determine the molecular, pharmacological and toxicological potential of new and existing drugs and to identify and characterize drug-induced protein-protein interactions in detail. Preclinical *in vitro* screening systems are essential for the development and discovery of new drugs but major limitations, such as high costs, low throughput, and limitations with respect to specificity and sensitivity, still exist. For this reason, new innovative screening systems are crucial to be able to identify new therapeutic drugs, their potential combinations with existing drugs and drug-induced protein-protein interactions.

In view of the crucial role of human peroxisome proliferator-activated receptor gamma (PPARγ) in the development of several obesity-related cancers and as a potential therapeutic target for autoimmune and inflammatory diseases 15-17, we developed an improved Clover/mRuby2-based flow cytometry-based FRET assay which proved to be suitable for determination of both protein-protein interactions and alterations in protein binding intensity and affinity upon drug treatment of living cells.

The ligand‑dependent, activated transcription factor, PPARγ belongs to the nuclear hormone receptor (NHR) superfamily and plays a crucial role in the development of several human diseases and as a therapeutic target 15-18. It is subdivided into four isoforms 19, 20. PPARγ1 is expressed in nearly all tissues, including heart, muscle, colon, kidney, pancreas, and spleen 21-25, whereas PPARγ2 is mainly expressed in adipose tissues 26-28. The PPARγ isoform 3 was identified in macrophages, large intestine, and white adipose tissues and isoform 4 in endothelial cells 19, 20. PPARγ possesses a central deoxyribonucleic acid (DNA)-binding domain that recognizes sequence‑specific PPAR response elements (PPREs) in the promoter region of target genes 26, 29. After activation by a tissue‑ and natural or synthetic ligand‑specific stimulus, PPARγ is translocated to the nucleus, where it changes its structure and regulates gene transcription which is important for cell differentiation, various metabolic, physiological and pathophysiological processes 30-34.

The PPARγ-regulated transcriptional activation of target genes is a complex multistep process and depends on the binding or not of a cognate ligand to the receptor. The process is achieved by heterodimerization of PPARγ with RXRα, binding to PPREs and finally the recruitment of co-factors and other nuclear co-regulatory proteins 35-39. PPARγ acts as a ligand-dependent regulator of transcription and this depends on its ability to interact with co-regulator proteins but it can also act in an unbound manner 40. PPARγ can also bind directly to other proteins and inhibits signal transduction. This capability, called transrepression, is mainly mediated by direct protein-protein interactions between PPARγ and other transcription factors 41-43. In this way, PPARγ inhibits pro-inflammatory signalling and induces an anti-inflammatory response 44, 45. Typically, activation of the PPARγ-RXRα heterodimer by a PPARγ agonist triggers conformational changes in the receptor which releases the co-repressor complex and PPARγ recruits co-factor complexes or co-activators, such as steroid receptor co-activator 1 (SRC1), SRC3 and cyclic adenosine monophosphate response element binding protein (CBP)/p300 to the promoter region of target genes to initiate transcription 46-48. These, in turn, assemble a multi-component complex that stimulates transcription both through direct interaction with the core transcription machinery and through the acetylation of histone tails that make the adjacent chromatin transcriptionally competent. Subsequently, co-repressors are recruited to the DNA-bound PPARγ to nucleate the assembly of the repressor complex.

An important PPARγ co-repressor is N-CoR2, which plays a crucial role in adipocyte differentiation and regulation of adipogenesis, insulin sensitivity and type 2 diabetes mellitus 49, 50. N-CoR2 consists of N-terminal repressor domains (RDs) that can associate with histone deacetylases and SWI3 - adenosine deaminase 2 (ADA2) - N-CoR2 - transcription factor IIIB (TFIIIB) (SANT) domains that target the histone deacetylases, as well as two C-terminal interaction domains (IDs, ID1, ID2 and depending on the cell type there could be an additional ID3) that contain co-repressor nuclear receptor (CoRNR) box (NHR box) motifs 51-53. Sequences within and outside these motifs mediate the interaction between co-repressors and many NHRs, including PPARγ, in which NHRs preferentially interact with the different IDs 54, 55. Therefore, alternative splicing of single IDs themselves or between them is a crucial source of variability when functioning as interaction partner. N-CoR2 is recruited by PPARγ in the absence of an agonist or the presence of an antagonist of PPARγ and represses target gene expression by recruiting a histone deacetylase complex until an agonist triggers its disassembly accompanied by recruitment of co-activators 56-61. The binding of the agonist to PPARγ results in a conformational change in the receptor, leading to loss of co-repressor binding and subsequent recruitment of co-activators. PPARγ can interact with N-CoR2 in solution, but in some contexts it is only a weak repressor, perhaps because the co-repressor binding affinity of PPARγ is weaker than that of other NHRs and is modulated by DNA binding 57, 62-67.

In this report, a systematically improved flow cytometry-based FRET assay for the molecular verification of specific protein-protein interactions of NHR (PPARγ isoform 1 hereinafter referred as PPARγ1) and co-factors (RXRα and N-CoR2) in living cells is described. This combines, for the first time, the identification and characterization of binding intensity and affinity. Thus, by improving sensitivity, this assay system can be used for the identification of novel therapeutic targets for treatment of human diseases and for the detailed characterization of the binding profile of drugs.

## Methods

### Chemicals and reagents

All chemicals and reagents were of the highest grade of purity and when not indicated otherwise, commercially available from AppliChem GmbH (Darmstadt, Germany), Carl Roth GmbH (Karlsruhe, Germany), Merck KGaA (Darmstadt, Germany) and Sigma-Aldrich Chemie GmbH (Schnelldorf, Germany). The PPARγ antagonist, GW9662 68, was acquired from Cayman Chemical Company (Ann Arbor, USA) and the PPARγ agonist, rosiglitazone, from Enzo Life Sciences GmbH (Lörrach, Germany). Cell culture medium and supplements were purchased from PAA Laboratories GmbH (Cölbe, Germany) and Sigma-Aldrich Chemie GmbH.

### Cell culture

Human HEK293T cells 69 and murine J774A.1 macrophages (ATCC^®^ TIB-67^TM^) 70 were obtained from LGC Standards GmbH (Wesel, Germany). HEK293T cells were cultured in Dulbecco's Modified Eagle's Medium (DMEM) and J774A.1 cells in Roswell Park Memorial Institute (RPMI)-1640 in a humidified 5% carbon dioxide (CO_2_) atmosphere at 37 °C. Cell culture medium contained 10% (v/v) heat-inactivated fetal calf serum (FCS), 100 units/ml penicillin and 100 µg/ml streptomycin. The medium was changed three times a week and cells passaged before reaching confluency. When using dimethyl sulfoxide (DMSO), in all cases, the final concentration of DMSO did not exceed 0.1%, a concentration that was not cytotoxic to the cells.

### Generation and cloning of expression vectors

Constructs were generated by inserting the coding sequences of Clover, mRuby2, Clover-PPARγ1, mRuby2-RXRα and N-CoR2-mRuby2 into pHR´SIN-cPPT-SE by replacing the EGFP of the vector. Therefore, the coding regions of Clover, mRuby2 and by a 63bp-linker fused Clover-mRuby were amplified by polymerase chain reaction (PCR) from pcDNA3-Clover (Addgene#40259), pcDNA3-mRuby2 (Addgene#40260) and pcDNA3.1-Clover-mRuby2 (Addgene#49089) elongated with the additional bases of 5´-CGCCCGGGGGGGATCGCCGCCACC-3´ on their 5´ end and 5´-CCTGCAGGCATGCAA-3´ on their 3´ end for subsequent recombination into the BamHI-SbfI linearized target vector by In-Fusion^®^ HD cloning (Clontech Laboratories, Inc., Mountain View, USA). Fusion constructs of Clover-PPARγ1, mRuby2-RXRα and N-CoR2-mRuby2 were generated by multiple fragment cloning via the same approach. The single genes were amplified, whereas both primers for the amplification of the fluorophore had an additional base overhang, one a homologue of the vector and one of its fusion partner genes. In contrast, only one primer for the amplification of the transcription factors/repressor had an overhang for the recombination directly with the vector. Thus, pDsRed-Monomer-C1-PPARγ1 71 and pEGFP-C1-RXRα 72 were used as template DNA for full length human PPARγ1 and human RXRα, whereas a 5´-truncated human N-CoR2 was amplified from HEK293T cell cDNA (GenBank accession number MH507335.1), which contains only the ID2 and not the ID1 exon (ID2/ΔID1 exon), using 5´- ATGAGCGTCCTCGAGAGGCAAA-3´ as 5´-primer. The coding sequences of the single genes were fused together by In-Fusion^®^ HD cloning, additionally amplified as one gene fragment and then inserted into the target vector.

Since HEK293T do not have the complete exon containing the ID1 box motif, the whole ID1 exon of the sequence 5´-GCCTTATGACCTATAGAAGCCAGGCGGTGCAGGAACATGCCAGCACCAACATGGGGCTGGAGGCCATAATTAGAAAGGCACTCATGG-3´ was synthesized by IDT - Integrated DNA technologies, Inc. (Coralville, USA), amplified and fused into the vector construct of N-CoR2 ID2/∆ID1 exon-mRuby2 to generate N-CoR2-mRuby2. For the generation of the different N-CoR2 mutants, ∆CoRNR box ID2 and ∆CoRNR box ID1 the appropriate DNA sequences 5´-CTGGCCCAGCACATCAGTGAGGTCATC-3´ (for CoRNR box ID2) and 5´-CTGGAGGCCATAATTAGAAAGGCACTC-3´ (for CoRNR box ID1) were deleted out of the N-CoR2 sequence by site directed mutagenesis. The same approach was used for the generation of mRuby2-RXRα Δ414-462. Genbank accession numbers are: Clover (JX489388); mRuby2 (JX489389); human PPARγ1 (NP_005028.4); human RXRα (NP_002948.1), N-terminal truncated human N-CoR2∆1-1105; 2310-2338 (NP_001070729.2).

### Transfection, lentiviral transduction and cell sorting

To generate infectious lentiviral particles for transduction of expression vectors, 2 × 10^6^ HEK293T cells were seeded into 10 cm dishes and cultured overnight, as described above, to allow attachment of HEK293T cells. The next day, HEK293T cells were transfected using the JetPRIME^TM^ transfection reagent (PEQLAB Biotechnologie GmbH, Erlangen, Germany), as described by the manufacturer. Transfection was carried out with 1.5 µg of the second-generation lentiviral packaging plasmid psPAX2 (Addgene#12260), 0.5 µg of the envelope expressing plasmid pMD2.G (Addgene#12259) and 2 µg of the cloned expression vector, encoding for the gene of interest. After 4 h, transfection medium was replaced and HEK293T cells were cultured in fresh growth medium for another 24 h to 48 h. Gene activity was analyzed by fluorescence microscopy. Twenty-four to 48 h post transfection the supernatant containing infectious lentiviral particles was harvested, centrifuged (500 x *g*, 4 °C, 5 min) and filtered through a 0.2 μm filter (Merck KGaA). The Lenti-X^TM^ Concentrator reagent (Takara Bio Europe SAS, Saint-Germain-en-Laye, France) was used as described by the manufacturer for concentrating lentiviral stocks. For transduction, 1 x 10^5^ of target HEK293T or J774A.1 cells were incubated in 6 well plates for 24 h to 48 h in cell culture medium containing concentrated infectious lentiviral particles. Subsequently, the medium was changed daily for at least ten days. Afterwards HEK293T cells were sorted for green- and/or red-positive cells using the BD FACSAria*™* III cell sorter (BD Biosciences, Heidelberg, Germany) and the FACS Diva software (BD Biosciences, Heidelberg, Germany).

### PPARγ‑dependent transactivation assay of HEK293T cells

For the PPARγ‑dependent transactivation assay, 1 × 10^4^ HEK293T cells per well were seeded into 96‑well plates and cultured overnight, as described above, to allow attachment of the HEK293T cells. The next day, the HEK293T cells were transiently transfected using the JetPRIME^TM^ transfection reagent (PEQLAB Biotechnologie GmbH), as described by the manufacturer. Transfection was carried out with 0.01 µg of the cloned expression vector(s), encoding the gene(s) of interest, 0.09 µg per well p(AOX)_3_‑TK‑Luc (89, 90) and 0.0005 µg per well pRL‑CMV (Promega GmbH, Mannheim, Germany). After 4 h, the transfection medium was replaced and the HEK293T cells were cultured in fresh growth medium for another 24 h.

The PPARγ‑dependent transactivation assay was based on the PPRE containing reporter vector p(AOX)_3_‑TK‑Luc. The cloned expression vector(s), encoding for the gene(s) of interest were co‑transfected, as described above, in combination with the control vector pRL‑CMV, encoding *Renilla* luciferase, to normalize *Firefly* luciferase activity for transfection efficiency. Following transfection, the cells were incubated with 1 µM rosiglitazone and 10 μM GW9662 for 24 h alone or both in combination. The PPARγ‑dependent transactivation assay in HEK293T cells, using a 96‑well plate format, was performed in quadruple. Transactivation was analyzed using a 96‑well plate format in a Mithras LB940 multimode reader (Berthold Technologies, Bad Wildbad, Germany) or an EnSpire^®^ Multimode Plate Reader (Perkin Elmer, Inc., Waltham, USA).

### Western blot analysis

HEK293T cells were resuspended in lysis buffer and sonicated. After centrifugation (15,000 x *g*, 4 °C, 5 min), the protein concentration was determined in the lysate by a Lowry protein assay kit (Bio-Rad Laboratories GmbH, Munich, Germany). For immune detection of Clover, mRuby2, Clover fused (63bp) mRuby2, Clover-PPARγ1, mRuby2-RXRα, mRuby2-RXRα Δ414-462 and N-CoR2-mRuby2 constructs, 100 µg protein per sample was separated on 10% sodium dodecyl sulfate (SDS) polyacrylamide gels followed by transfer onto nitrocellulose membranes (both Bio-Rad Laboratories GmbH) basically following standard procedures. Subsequently, membranes were incubated with first antibodies against GFP (1:1,000; ab1218, Abcam plc, Cambridge, UK), human PPARγ (1:1,000; D69, Cell Signaling Technology, Danvers, USA), human RXRα (1:1,000; NB100-1466, Novus Biologicals, LLC, Littleton, USA), human N-CoR2 (1:1,000; ab2781, Abcam plc) and tRFP (1:1,000; AB233, Evrogen Joint Stock Company, Moscow, Russia) followed by Alexa Fluor^®^ 488, 546 or 647 (Life Technologies Inc., Carlsbad, USA) fluorescent dyes secondary antibodies (1:10,000). Blots were visualized using the ChemiDoc XRS+ system (Bio-Rad Laboratories GmbH).

### Fluorescence microscopy

For fluorescence microscopy, 2 x 10^4^ HEK293T cells per well were seeded on all-in-one 8 wells chamber slides. Cultivation continued for 24 h, as described above, to allow attachment of the HEK293T cells, followed by fixation of cells by incubation with Roti^®^-Histofix solution (Carl Roth GmbH) combined with concomitant counterstaining of cell nuclei with 1 μg/ml Hoechst 33342 dissolved in DMEM in the dark at 37 °C for 15 min. Subsequently, cells were washed twice with DMEM. The cells were excited with a laser at λ_ex_ = 405 nm, 488 nm and 561 nm. Clover, mRuby2 and Hoechst 33342 were determined on the chamber slides using a 40x immersion water objective with a Zeiss confocal laser scanning microscope (CLSM) 510 Meta (Carl Zeiss AG, Jena, Germany). Uniform laser conditions regarding master and digital gains, pinholes and laser intensities were used for fluorescence detection among the fluorophores alone and for fluorophores assigned to different cellular functional proteins.

### Quantification of subcellular co-localization

Correlation analysis of subcellular co-localization was performed using the open source image processing program ImageJ with the plugin “JACoP” 72. For this, areas of single whole cells or nuclei were framed in brightfield or Hoechst-stained images and the Pearson´s correlation coefficient (PCC) of these regions were calculated from the images, requiring the use of the 488 nm and 561 nm laser. The single R-values obtained were transformed into Fisher z-values, which converts correlations into interval scales values. From these data means, standard deviation (SD) and significances were calculated and further separately transformed back into R-values.

### Fluorescence lifetime imaging microscopy (FLIM)

The fluorescence lifetime of the fluorescent proteins depends on their microenvironment. Intermolecular interactions will change the fluorescence lifetime. Thus, the FLIM method was used to observe the capacity of the proteins to generate a FRET signal inside the cells. The cell images were observed with a FluoView^TM^ FV1000 CLSM microscope (Olympus Europa SE & Co. KG, Hamburg, Germany) with FLIM-extension (PicoQuant, Berlin, Germany). The cells were excited with a 1 ns pulse laser at λ_ex_ = 440 nm for fluorescence lifetime measurements.

HEK293T cells were seeded on glass coverslips 24 h before measurement and were viewed directly on the coverslip using a 60x immersion oil objective. All images were obtained from living cells to prevent changes in the protein structure or its microenvironment during the procedure of cell fixing. The result was taken as a standard reference for flow cytometry-based FRET analyses.

### Flow cytometry measurements

**Figure 1:**
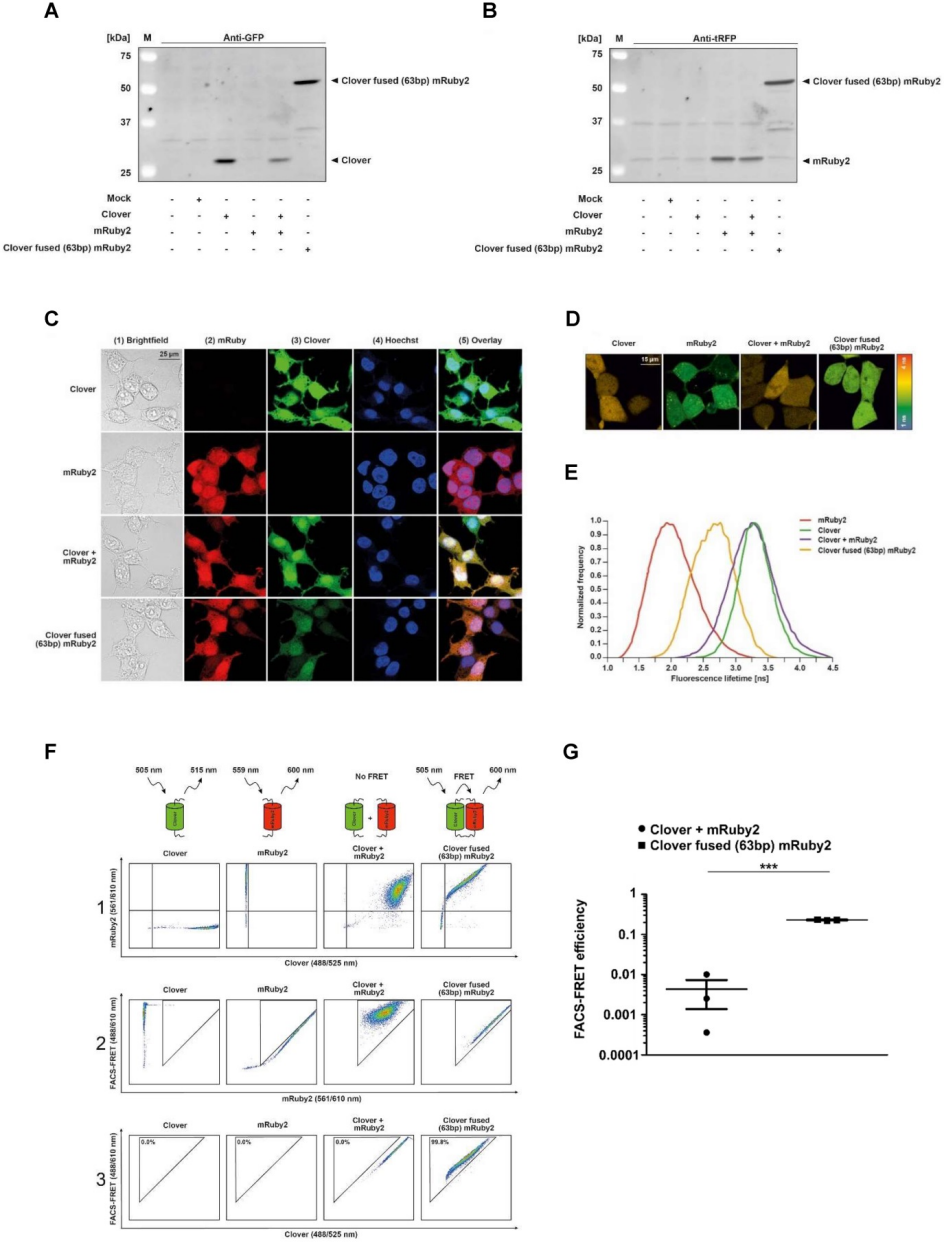
** Experimental setup and gating strategy of FRET measurements.** Clover, mRuby2, Clover + mRuby2 and Clover fused (63bp) mRuby2 protein expression are shown in total HEK293T cell lysates by Western blot analysis against GFP **(A)** and tRFP **(B)**. Fluorescence microscopy images **(C)** of Clover, mRuby2, Clover + mRuby2 and Clover fused (63bp) mRuby2 expressing HEK293T cells. Panel 1 (1) shows the brightfield. Cells expressing mRuby2 are depicted in panel 2 (2), Clover-positive cells in panel 3 (3). Cell nuclei were counterstained with Hoechst 33342 (panel 4 (4)). An overlay to estimate cytosolic and nuclear region is provided in panel 5 (5). FLIM images **(D)** of HEK293T cells with the controls Clover, mRuby2, Clover + mRuby2 and Clover fused (63bp) mRuby2. The histogram **(E)** shows the normalized frequency of fluorescence lifetimes in the images. Experimental setup and gating strategy to measure FRET by flow cytometry are depicted in living cells **(F)**. HEK293T cells were stably transduced with the controls Clover, mRuby2, Clover + mRuby2 as well as the Clover fused (63bp) mRuby2 and analyzed using a flow cytometer. Double positive cells were gated in panel 1 (1). False-positive FRET signals resulting from mRuby2 excitation by the 488 nm laser were excluded in panel 2 (2). In panel 3 (3), the remaining cells were evaluated for FRET by adjusting a gate defining cells which were co-transduced with Clover + mRuby2 and should be FRET-negative. The numbers in panel 3 (3) give total percentages of FRET-positive HEK293T cells. Images and flow cytometry-plots are representative for experiments which were performed at least three times. **(G)** FRET efficiencies were determined as described in Materials and Methods by analyzing Clover/mRuby2-double positive cells only. Experiments were performed at least three times. ***P ≤ 0.001.

Flow cytometry-based FRET measurements were performed using a FACSFortessa (BD Bioscience) equipped with standard 488 nm and 561 nm lasers. To measure Clover and FRET, cells were excited with the standard 488 nm laser and fluorescence was detected in the green channel with a standard 525/50 nm filter, while the FRET signal was measured with a standard 610/20 nm filter in the channel. The mRuby2 cells were excited with the standard 561 nm laser, while emission was also detected with a standard 610/20 nm filter. For each sample, we analyzed a minimum of 10,000 Clover- and/or mRuby2-positive cells according to the gating strategy shown in **Figure 1F**. Analyses of flow cytometric data were performed using the FlowJo software (Version X).

### Flow cytometry-based FRET efficiency

Following appropriate gating of Clover/mRuby2-double positive cells, flow cytometry-based FRET efficiencies were calculated based on fluorescence medians of Clover (488/525 nm), FRET (488/610 nm), and mRuby2 (561/610 nm) using the FRET calculator protocol provided by Ujlaky-Nagy et al. 11 with reference to our FLIM control setting. Single-positive as well as fluorescence negative cells served as background controls. Gating strategies are depicted in **Supplementary Figures 1**, **4**, **6**, **7, 8, and 9**.

### Statistics

All data are presented as means ± SD. Each experiment was performed at least three times. Statistical analysis was done either with one- or two-way-analysis of variance modified with Bonferroni's multiple comparison test or unpaired and paired Student's *t*-test, respectively. Differences were considered significant at: *P ≤ 0.05, **P ≤ 0.01 and ***P ≤ 0.001.

## Results

### Detection of Clover, mRuby2 and Clover fused (63bp) mRuby2 protein expression

To confirm that the fluorophore probes to be used in this study were actually expressed in the cells used, the protein expression of the fluorophores Clover, mRuby2 and Clover fused with a linker sequence to mRuby2 (Clover fused (63bp) mRuby2) in human embryonic kidney 293 T (HEK293T) cells was verified by Western blot analysis. Stained with an antibody against GFP, Clover was only detected in Clover-expressing HEK293T Clover, HEK293T Clover + mRuby2 and HEK293T Clover fused (63bp) mRuby2 cells (**Figure 1A**). In turn, when stained with an antibody against tRFP, only in mRuby2-expressing HEK293T mRuby2, HEK293T Clover + mRuby2 and HEK293T Clover fused (63bp) mRuby2 cells mRuby2 was observed (**Figure 1B**). Neither when stained with an antibody against GFP nor with an antibody against tRFP was the protein expression of Clover, mRuby2 or Clover fused (63bp) mRuby2 detected in wild type (WT) and Mock transfected HEK293T cells.

### Determination of the cellular localization of Clover, mRuby2 and Clover fused (63bp) mRuby2 by fluorescence microscopy

To determine the cellular localization of the fluorophores used, corresponding Clover- and mRuby2-expressing HEK293T cells were mounted on all-in-one chamber slides and fixed. To distinguish between cytosolic and nuclear localization, Hoechst 33342 was used as a nuclear counterstain. As shown in **Figure 1C**, mRuby2 (**panel 2**), Clover (**panel 3**) and Clover fused (63bp) mRuby2 were evenly distributed throughout the cell.

### Validation of Clover, mRuby2 and Clover fused (63bp) mRuby2 for flow cytometry FRET measurements by FLIM

FLIM was used to determine the different fluorescence lifetimes of the intracellular fluorescent proteins. HEK293T cells were transduced to stably express Clover/mRuby2 alone or in combination and Clover fused (63bp) mRuby2 in order to investigate the potential FRET activity of the Clover fused (63bp) mRuby2 protein. As shown in **Figure 1D** and **1E**, the fluorescence lifetime of Clover was highest with a frequency of 3.4 ns ± 0.1 ns, whereas the most frequent fluorescence lifetime of mRuby2 was 1.7 ns ± 0.1 ns. In cells that expressed both Clover and mRuby2 as single proteins, only the fluorescence lifetime of Clover was detectable. In contrast, cells expressing Clover fused (63bp) mRuby2 showed a most frequent fluorescence lifetime of 2.7 ns ± 0.3 ns. This reduction in the fluorescence lifetime from 3.4 ns ± 0.1 ns to 2.7 ns ± 0.3 ns resulted from changes in FRET activity. FRET efficiency of the fusion protein can be calculated from the lifetime of the construct and of the free Clover, roughly resulting in a value of 0.23. This absolute measure of FRET is considered in calculating the flow cytometry-based FRET efficiencies.

### Establishment of Clover, mRuby2 and Clover fused (63bp) mRuby2 for FRET measurement by flow cytometry

In a previous study, Banning and colleagues established a flow cytometry-based FRET assay system to identify and analyze protein-protein interactions in living cells 8. For the analysis of interactions of PPARγ1 with its co-factor RXRα and its co-repressor N‑CoR2 in living cells, we adapted and extended this innovative method. We established a cytometry-based FRET assay with the FRET pair fluorophores Clover/mRuby2 14. For FRET measurements by flow cytometry, we first analyzed HEK293T cells stably expressing Clover/mRuby2 alone or in combination and Clover fused (63bp) mRuby2 (**Figure 1F**). We gated living cells to forward and side scatter (FSC/SSC, not shown) and compensated for Clover and mRuby2 to specifically evaluate FRET in double positive Clover + mRuby2 and Clover fused (63bp) mRuby2 cells. A triangular gate was introduced in panel 2 and false-positive FRET signals resulting from mRuby2 excitation by the 488 nm laser were excluded. A second triangular gate was introduced in panel 3, where FRET was plotted against Clover to determine FRET-positive cells. This gate was adjusted to FRET-negative HEK293T Clover + mRuby2 cells. Consequently, HEK293T Clover and HEK293T mRuby2 cells exhibited no FRET signal, whereas 99.8% of cells expressing Clover fused (63bp) mRuby2 fusion protein (**Figure 1F**, panel 3) showed a FRET signal. These cells were further used as a positive control. In contrast, 0.0% cells expressing Clover and mRuby2 (**Figure 1F**, panel 3) as individual proteins showed FRET, thus representing the internal negative control. To verify our data, we analyzed the FRET efficiency. As shown in **Supplementary Figure 1**, gating for Clover/mRuby-positive cells was performed. Fluorescence medians of Clover (488/525 nm), FRET (488/610 nm), and mRuby2 (561/610 nm) were used to calculate FRET efficiency, including medians of fluorescence negative HEK293T cells as background control (**Figure 1G**), supporting the information from the raw flow cytometric data shown in **Figure 1F**.

### Verification of Clover-PPARγ1, mRuby2-RXRα and mRuby2-RXRα Δ414-462 protein expression

**Figure 2:**
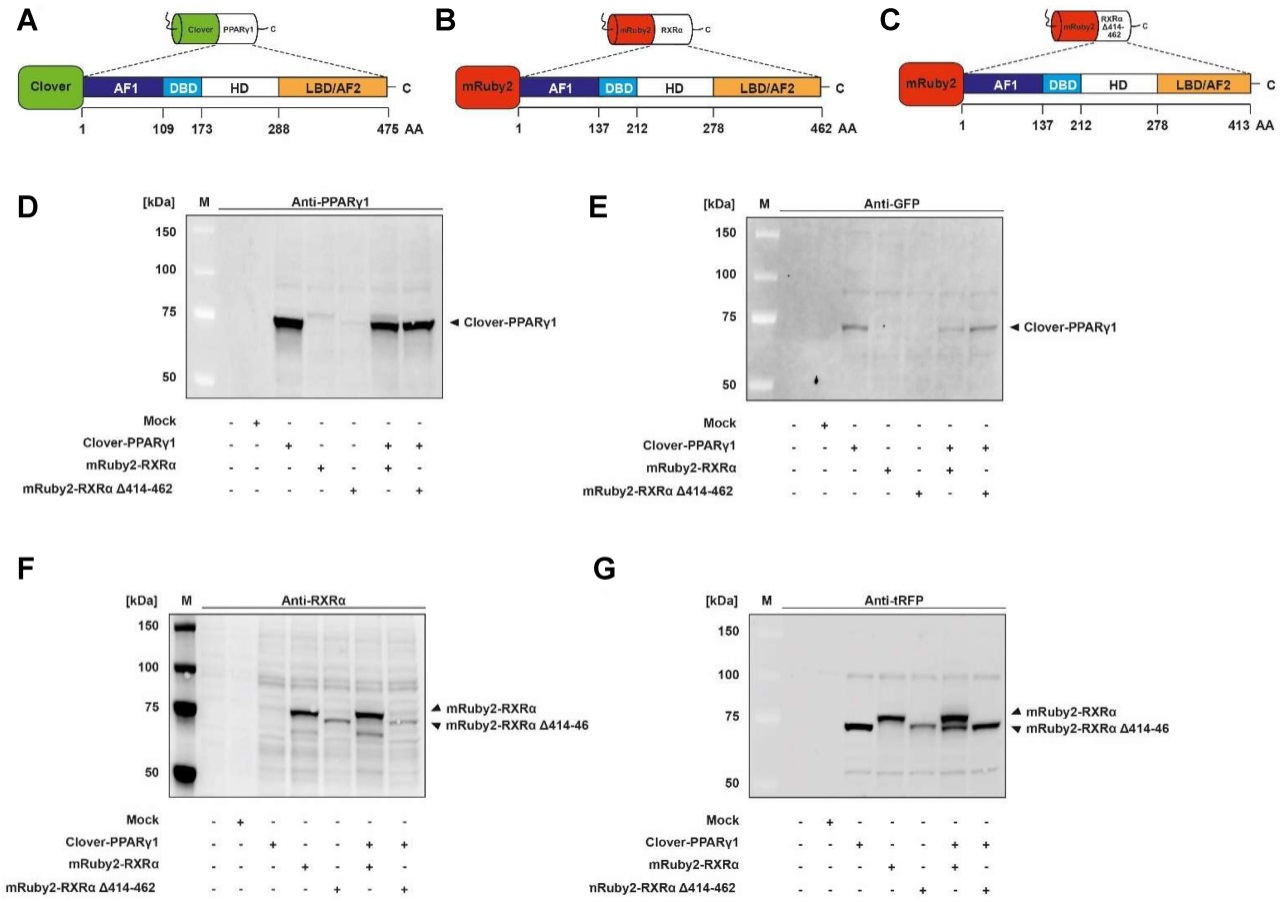
** Determination of Clover-PPARγ1, mRuby2-RXRα and mRuby2-RXRα Δ414-462 protein expression.** Graphical scheme of the structure of the full length human PPARγ1 protein **(A)**; the full length human RXRα protein** (B)** and the human RXRα deletion construct with a C-terminal absence of the sequence AA 414-462 **(C)**. PPARγ1 was N-terminally labeled with the green fluorophore Clover and both of the two RXRα constructs N-terminally with the red fluorophore mRuby2. Clover-PPARγ1, mRuby2-RXRα and mRuby2-RXRα Δ414-462 protein expressions are shown in total HEK293T cell lysates visualized by Western blot analysis against human PPARγ **(D)**, GFP **(E)**, human RXRα **(F)** and tRFP **(G)**. Images are representative of experiments which were performed at least three times.

Expression of Clover-PPARγ1 (**Figure 2A**), mRuby2-RXRα (**Figure 2B**) and mRuby2-RXRα Δ414-462 (**Figure 2C**) fusion proteins in HEK293T cells was verified by Western blot analysis. Specific antibodies against GFP, tRFP, human PPARγ and human RXRα were used. As shown in **Figure 2D** and **2E**, the Clover-PPARγ1 fusion protein was detected in cells when stably expressing Clover-PPARγ1, Clover-PPARγ1 + mRuby2-RXRα and Clover-PPARγ1 + mRuby2-RXRα Δ414-462. In the literature it is well established that due to the deletion of the C-terminus of RXRα and the associated lack of the amino acid (AA) sequence 414 to AA 462, RXRα Δ414-462 significantly reduces binding to PPARγ 68. The protein molecular weight of this deletion construct was 5.7 kDa smaller than the complete mRuby2-RXRα fusion protein (**Figure 2F** and **2G**). Both constructs were only detected in cells expressing mRuby2-RXRα, mRuby2-RXRα Δ414-462, Clover-PPARγ1 + mRuby2-RXRα and Clover-PPARγ1 + mRuby2-RXRα Δ414-462. WT and Mock transfected HEK293T cells were used as negative controls for expression of Clover-PPARγ1 (**Figure 2D** and **2E**), mRuby2-RXRα (**Figure 2F** and **2G**) and mRuby2-RXRα Δ414-462 (**Figure 2F** and **2G**). Our analysis at the protein level by Western blot, depicted in **Figure 2G**, indicates that the anti-tRFP antibody also binds to Clover, allowing detection of a specific band at the level of Clover-PPARγ1. This band is very prominent in co-transduced HEK293T Clover-PPARγ1 + mRuby2-RXRα cells and thus, shows clearly the simultaneous protein expression of the two fusion proteins in these cells. Due to the deletion of 48 AA (Δ414-462) of the PPARγ1 ID of RXRα, Clover-PPARγ1 and mRuby2-RXRα Δ414-462 are approximately equal in size. For this reason, only one band is visible in co-transduced HEK293 Clover-PPARγ1 + mRuby2-RXRα Δ414-462 cells.

### Mechanistic functionality of Clover, Clover-PPARγ1, mRuby2, mRuby2-RXRα and mRuby2-RXRα Δ414-462

To examine whether PPARγ-dependent transactivation is altered, we performed a set of experiments using a PPRE-dependent reporter system. HEK293T cells expressing Clover, Clover-PPARγ1, mRuby2, mRuby2-RXRα and mRuby2-RXRα Δ414-462 were treated for 24 h with 1 µM of the PPARγ agonist, rosiglitazone and 10 µM of the irreversible binding PPARγ antagonist, 2-chloro-5-nitrobenzanilide (GW9662) 68 or a combination of both. *Firefly* and control *Renilla* luciferase luminescence values were determined in each sample. The ratios of *Firefly* to *Renilla* luciferase luminescence were used for normalization.

**Figure 3:**
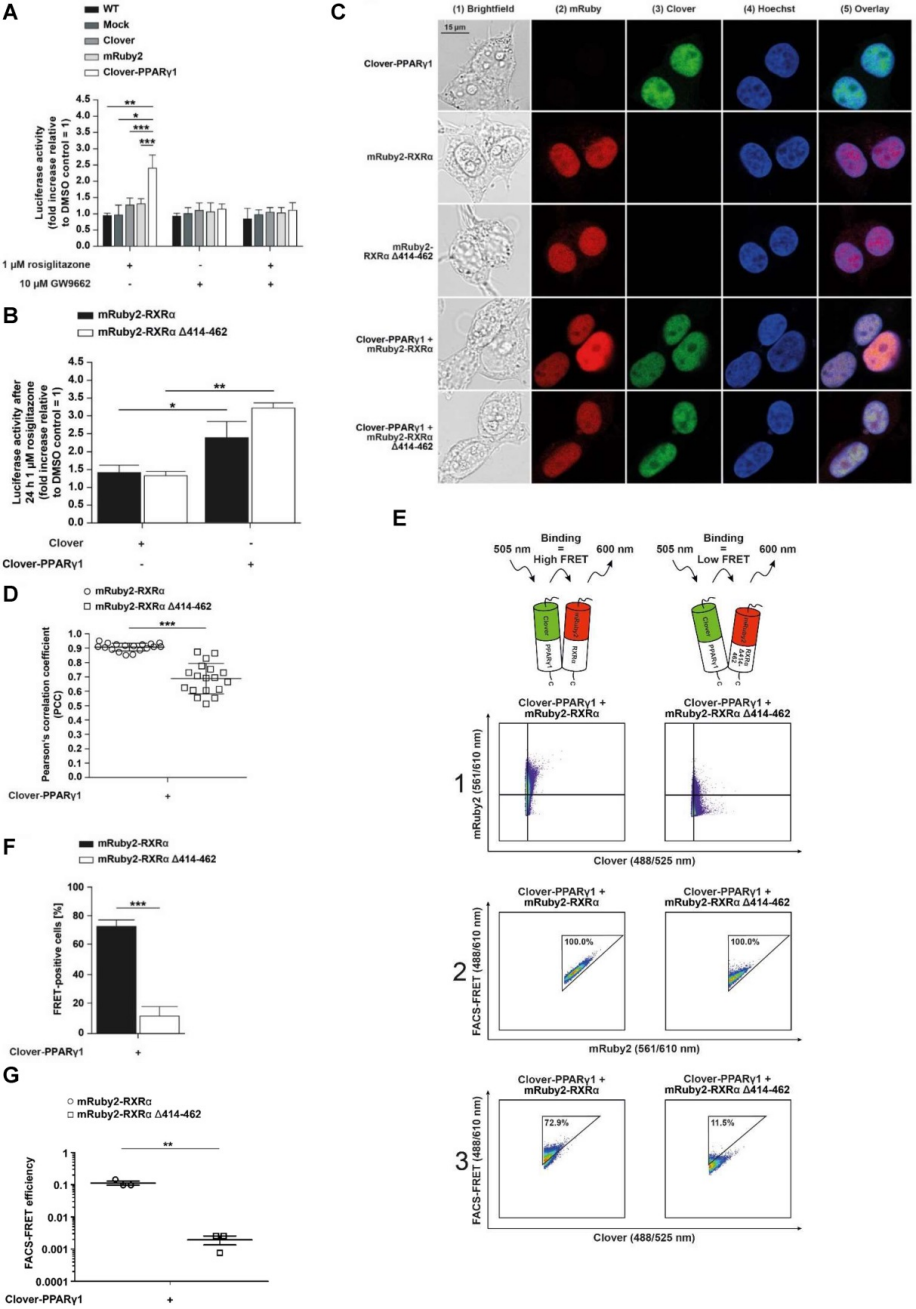
** Analysis of protein-protein interactions between Clover-PPARγ1, mRuby2-RXRα and mRuby2-RXRα Δ414-462.** A PPARγ‑dependent transactivation assay was used in HEK293T cells to verify the mechanistic functionality of the Clover, Clover-PPARγ1, mRuby2, mRuby2-RXRα and mRuby2-RXRα Δ414-462 **(A** and** B)**. HEK293T cells expressing protein(s) as indicated were stimulated for 24 h with 1 µM rosiglitazone and 10 µM GW9662 alone or in combination. Values from PPARγ‑dependent transactivation experiments are means ± SD of three to ten individual experiments. Each PPARγ‑dependent transactivation assay experiment was performed in quadruple. *P ≤ 0.05, **P ≤ 0.01 and ***P ≤ 0.001. Fluorescence microscopy images of HEK293T cells expressing Clover-PPARγ1, mRuby2-RXRα, mRuby2-RXRα Δ414-462, Clover-PPARγ1 + mRuby2-RXRα and Clover-PPARγ1 + mRuby2-RXRα Δ414-462 are depicted **(C)**. The brightfield is shown in panel 1 (1). The mRuby2-positive cells are depicted in panel 2 (2), Clover in panel 3 (3), Hoechst 33342-stained cell nuclei in panel 4 (4) and an overlay to estimate the localization is provided in panel 5 (5). Images are representative for experiments which were performed at least three times. The PCC was used for the correlation quantification of the subcellular co-localization of Clover-PPARγ1 expressed in combination with mRuby2-RXRα or mRuby2-RXRα Δ414-462 **(D)**. R-Values are means ± SD of at least 18 individual cells. ***P ≤ 0.001. Representative primary flow cytometry-plots from an experiment which was performed three times showing the amount of FRET-positive living HEK293T cells expressing Clover-PPARγ1 + mRuby2-RXRα and Clover-PPARγ1 + mRuby2-RXRα Δ414-462 **(E)**. The numbers in panel 3 (3) give total percentages of HEK293T cells within the FRET gate. A comparison of the total percentages of FRET-positive living HEK293T Clover-PPARγ1 + mRuby2-RXRα and HEK293T Clover-PPARγ1 + mRuby2-RXRα Δ414-462 cells is depicted in **(F).** Flow cytometry-based FRET efficiencies, depicted in **(G)**, are calculated as described in Materials and Methods. Values are means ± SD of three experiments. **P ≤ 0.01, ***P ≤ 0.001.

In line with its published action, rosiglitazone alone showed significant agonistic effects and led to an approximately 2.5‑fold PPARγ‑transactivation after 24 h stimulation in Clover-PPARγ1 cells (**Figure 3A**). In HEK293T WT and Mock transfected cells as well as HEK293T cells expressing Clover and/or mRuby2, rosiglitazone and GW9662 alone or in combination showed no agonistic effects and led to an approximately 1.25‑fold higher transactivation of PPARγ compared to DMSO control treatment (**Supplementary Figure 2**).

There were no differences between Clover + mRuby2-RXRα and Clover + mRuby2-RXRα Δ414-462 cells in response to rosiglitazone (**Figure 3B**). In this case, a roughly 1.4‑fold higher transactivation of PPARγ compared to DMSO control treatment was detected. In contrast to this observation, in Clover-PPARγ1 + mRuby2-RXRα and Clover-PPARγ1 + mRuby2-RXRα Δ414-462 cells, a significantly increased 2.5‑fold to 3.25‑fold PPARγ‑transactivation was observed. Altogether, these results indicate that the Clover-PPARγ1 fusion protein is functional and responsive to drug treatment.

### Assessment of the fluorescence and cellular localization of Clover-PPARγ1, mRuby2-RXRα and mRuby2-RXRα Δ414-462 by fluorescence microscopy

Fluorescence microscopy was used to assess the cellular localization of Clover-PPARγ1, mRuby2-RXRα and mRuby2-RXRα Δ414-462 in HEK293T cells. Cell nuclei were visualized by Hoechst 33342-staining (**Figure 3C**,** panel 4**), confirming the mainly nuclear localization of Clover-PPARγ1 (**Figure 3C**, **panels 3** and **5**), mRuby2-RXRα (**Figure 3C**, **panels 2** and **5**) and mRuby2-RXRα Δ414-462 (**Figure 3C**, **panels 2** and **5**).

The PCC was used to assess the correlation between the subcellular co-localization of Clover-PPARγ1 with mRuby2-RXRα or mRuby2-RXRα Δ414-462. As shown in **Figure 3D**, significant differences in the PCC of HEK293T Clover-PPARγ1 + mRuby2-RXRα (0.912 ± 0.154) compared to HEK293T Clover-PPARγ1 + mRuby2-RXRα Δ414-462 (0.706 ± 0.226) were detected. Because of the overlapping spectra of the fluorophores Clover and mRuby2, a possible irradiation of fluorescence into each of the confocal microscopy channels was expected. Therefore, the PCC of cells expressing only single fusion proteins was also calculated. The highest background value was measured for Clover-PPARγ1 alone and was in the range of 0.7 (**Supplementary Figure 3**). Considering this value as background signal, mRuby2-RXRα Δ414-462 does not associate with Clover-PPARγ1, whereas the full-length RXRα seems to specifically co-localize (**Figure 3D**).

### Protein-protein interactions of Clover-PPARγ1 - mRuby2-RXRα and Clover-PPARγ1 - mRuby2-RXRα Δ414-462 determined by flow cytometry-based FRET

Using the established experimental setup and the gating strategy for FRET measurements by flow cytometry (**Figure 1F**), we analyzed the protein-protein interactions of Clover-PPARγ1 with mRuby2-RXRα and with the RXRα deletion construct mRuby2-RXRα Δ414-462 in living HEK293T cells to substantiate the functionality of our flow cytometry-based FRET assay system. In **Figure 3E**, a representative flow cytometry-plot is shown. Consequently, due to the deletion of the C-terminus of mRuby2-RXRα Δ414-462, which is necessary for PPARγ1-binding, cells expressing this deletion construct showed reduced binding to Clover-PPARγ1. In this case, only 11.5% of the double positive (**Figure 3E**, **right panel 1**, **upper right quadrant**) HEK293T cells expressing Clover-PPARγ1 and mRuby2-RXRα Δ414-462 showed a FRET signal (**Figure 3E**, **right panel 3**) whereas 72.9% of double positive (**Figure 3E**, **left panel 1**, **upper right quadrant**) HEK293T Clover-PPARγ1 + mRuby2-RXRα cells showed a FRET signal (**Figure 3E**, **left panel 3**). Overall, the percentage of FRET-positive HEK293T Clover-PPARγ1 + mRuby2-RXRα cells was significantly higher compared to HEK293T cells expressing Clover-PPARγ1 and mRuby2-RXRα Δ414-462. Hence, flow cytometry-based FRET gives a robust readout for the interaction of PPARγ1 with RXRα that is highly superior as compared to non-background corrected co-localization microscopy (**Figures 3D** vs **3F**). FRET efficiencies (**Figure 3G**) reflect FACS data. Appropriate gating is shown in **Supplementary Figure 4**.

### Functional expression of Clover-PPARγ1 and N-CoR2-mRuby2 constructs protein

**Figure 4:**
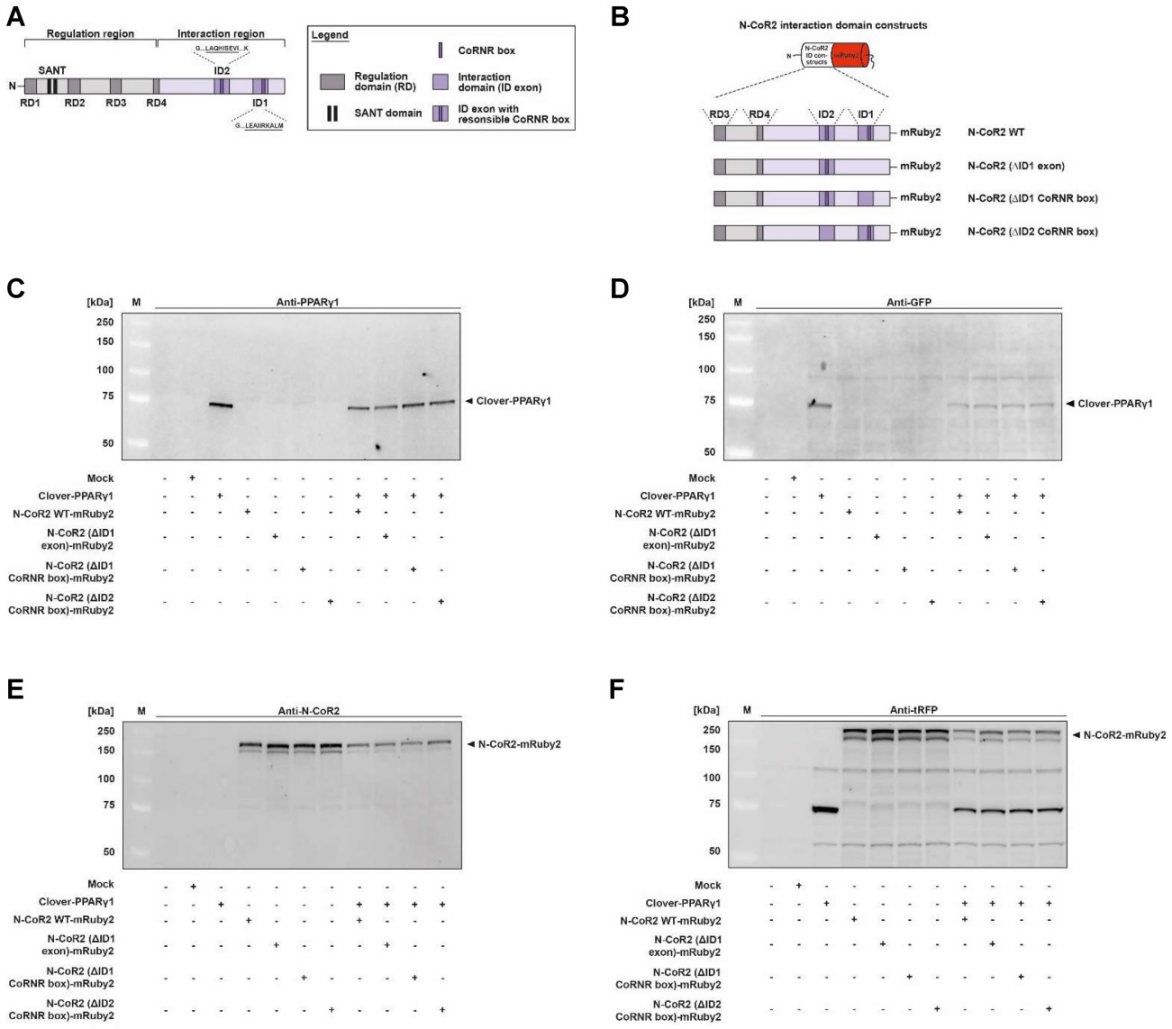
** Protein expression analysis of Clover-PPARγ1 and N-CoR2-mRuby2 constructs.** Graphical scheme of the structure of the full-length human N‑CoR2 protein **(A)** and the used N-CoR2 constructs, N-CoR2 WT-mRuby2, N-CoR2 (ΔID1 exon)-mRuby2 (amplified out of HEK293T cells cDNA), N-CoR2 (ΔID1 CoRNR box)-mRuby2 and N-CoR2 (ΔID2 CoRNR box)-mRuby2 **(B)**. All N-CoR2 constructs were C-terminally labeled with the red fluorophore mRuby2. Western blot analysis of total lysate of HEK293T cells stably expressing Clover-PPARγ1, N-CoR2 WT-mRuby2, N-CoR2 (ΔID1 exon)-mRuby2, N-CoR2 (ΔID1 CoRNR box)-mRuby2 and N-CoR2 (ΔID2 CoRNR box)-mRuby2 alone or in combination against human PPARγ1 (**C**), GFP **(D)**, human N-CoR2 (**E**) and tRFP **(F)**. Images are representative of experiments which were performed at least three times.

The protein expression of fusion proteins, Clover-PPARγ1, N-CoR2 WT-mRuby2 (**Figure 4A** and **4B**), N-CoR2 (ΔID1 exon)-mRuby2 (**Figure 4B**), N-CoR2 (ΔID1 CoRNR box)-mRuby2 (**Figure 4B**) and N-CoR2 (ΔID2 CoRNR box)-mRuby2 (**Figure 4B**), was determined by Western blot analysis. Clover-PPARγ1 was detected using an anti-PPARγ1 antibody (**Figure 4C**) and an anti-GFP antibody (**Figure 4D**) in HEK293T cells stably expressing this fusion protein. In cells stably expressing N-CoR2-mRuby2 constructs alone or in combination with Clover-PPARγ1, N-CoR2 WT-mRuby2, N-CoR2 (ΔID1 exon)-mRuby2, N-CoR2 (ΔID1 CoRNR box)-mRuby2 and N-CoR2 (ΔID2 CoRNR box)-mRuby2 could all be determined with an antibody against N-CoR2 (**Figure 4E**) and tRFP (**Figure 4F**). WT and Mock transfected HEK293T cells were used as negative controls for expression of Clover-PPARγ1 (**Figure 4C** and **4D**) and N-CoR2-mRuby2 constructs (**Figure 4E** and **4F**). As mentioned above, our analysis at the protein level by Western blot indicates that the anti-tRFP antibody also recognizes Clover, allowing detection of a specific band at the level of Clover- PPARγ1. This band was very prominent in HEK293T cells co-transduced with Clover-PPARγ1 + N-CoR2 constructs (**Figure 4F**), thus, showing clearly the simultaneous protein expression of the two fusion proteins in these cells.

### Mechanistic functionality of N-CoR2-mRuby2 constructs

To examine whether PPARγ-dependent transactivation is altered when the interaction domain of N-CoR2 is modified, we performed a set of experiments using a PPRE-dependent reporter system. HEK293T cells expressing Clover-PPARγ1 in combination with N-CoR2 WT-mRuby2, N-CoR2 (ΔID1 exon)-mRuby2, N-CoR2 (ΔID1 CoRNR box)-mRuby2 or N-CoR2 (ΔID2 CoRNR box)-mRuby2 were treated for 24 h with 1 µM rosiglitazone and compared to DMSO control treatment. *Firefly* and control *Renilla* luciferase luminescence values were determined in each sample. The ratios of *Firefly* to *Renilla* luciferase luminescence were used for normalization.

**Figure 5:**
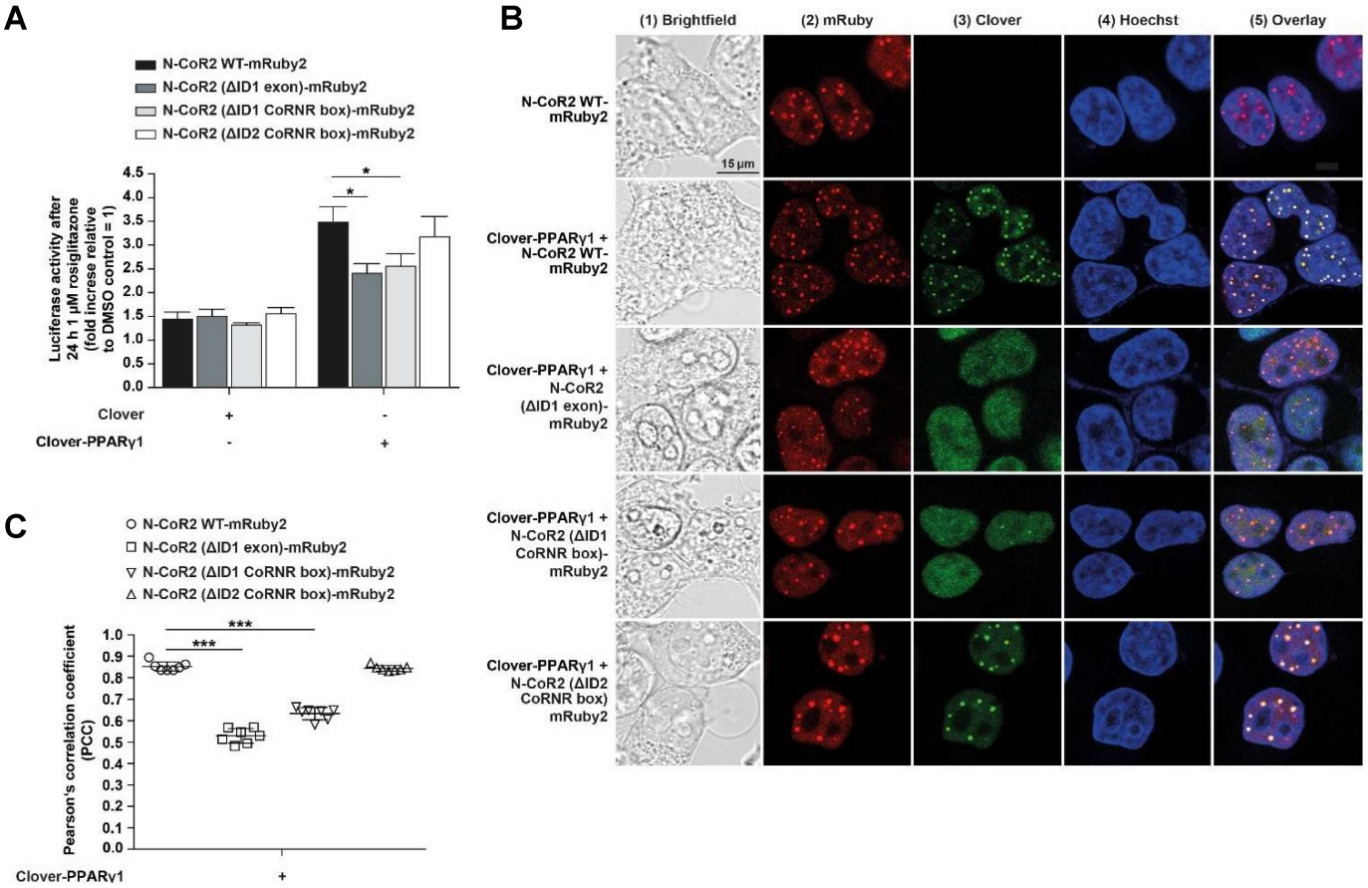
** Mechanistic functionality and cellular localization of Clover-PPARγ1 and N-CoR2-mRuby2 constructs.** A PPARγ‑dependent transactivation assay was used to verify the mechanistic functionality of HEK293T cells expressing Clover or Clover-PPARγ1 in combination with N-CoR2 WT-mRuby2, N-CoR2 (ΔID1 exon)-mRuby2, N-CoR2 (ΔID1 CoRNR box)-mRuby2 and N-CoR2 (ΔID2 CoRNR box)-mRuby2 **(A)**. HEK293T cells expressing protein(s) as indicated were stimulated for 24 h with 1 µM rosiglitazone. Values from PPARγ‑dependent transactivation experiments are means ± SD of four to 12 individually experiments. Each PPARγ‑dependent transactivation assay experiment was performed in quadruple. *P ≤ 0.05. Fluorescence microscopy images of HEK293T cells expressing N-CoR2 WT-mRuby2 and Clover-PPARγ1 in combination with N-CoR2 WT-mRuby2, N-CoR2 (ΔID1 exon)-mRuby2, N-CoR2 (ΔID1 CoRNR box)-mRuby2 and N-CoR2 (ΔID2 CoRNR box)-mRuby2 cells are depicted **(B)**. The brightfield is shown in panel 1 (1). The mRuby2-positive cells are depicted in panel 2 (2), Clover in panel 3 (3), Hoechst 33342-stained cell nuclei in panel 4 (4) and an overlay to estimate the localization is provided in panel 5 (5). Images are representative of experiments which were performed at least three times. The PCC was used for the correlation quantification of the sub-cellular co-localization of Clover-PPARγ1 expressed in combination with N-CoR2 WT-mRuby2, N-CoR2 (ΔID1 exon)-mRuby2, N-CoR2 (ΔID1 CoRNR box)-mRuby2 and N-CoR2 (ΔID2 CoRNR box)-mRuby2 in HEK293T cells** (C)**. R-Values are means ± SD of at least seven individual cells. ***P ≤ 0.001.

In Clover-PPARγ1 + N-CoR2 WT-mRuby2 cells, rosiglitazone showed agonistic effects and led to an approximately 3.5‑fold PPARγ‑transactivation (**Figure 5A**). Compared to Clover-PPARγ1 + N-CoR2 (ΔID1 exon)-mRuby2 and Clover-PPARγ1 + N-CoR2 (ΔID1 CoRNR box)-mRuby2 cells, the rosiglitazone-induced PPARγ‑transactivation was significantly increased. In Clover-PPARγ1 + N-CoR2 (ΔID2 CoRNR box)-mRuby2 cells, an approximately similar PPARγ‑transactivation as in Clover-PPARγ1 + N-CoR2 WT-mRuby2 cells was observed.

### Determination of the cellular localization of Clover-PPARγ1 and N-CoR2-mRuby2 constructs by fluorescence microscopy

A detailed fluorescence microscopic assessment of the cellular localization of HEK293T cells expressing fusion proteins of Clover-PPARγ1 (**panels 3** and **5**), N-CoR2 WT-mRuby2, N-CoR2 (ΔID1 exon)-mRuby2, N-CoR2 (ΔID1 CoRNR box)-mRuby2 or N-CoR2 (ΔID2 CoRNR box)-mRuby2 (**panels 2** and **5**) is shown in **Figure 5B**. The overlay (**panel 5**) showed a predominantly nuclear localization of Clover-PPARγ1 and N-CoR2-mRuby2 constructs. In all cases, when analyzing N-CoR2 constructs, strong red spots in the nuclei of cells were evident (here, only shown for N-CoR2 WT-mRuby2). However, in contrast to this unambiguous observation, spots like those seen in cells expressing N-CoR2 constructs were only observed for the Clover-PPARγ protein in a comparable variety and intensity in cells when either N-CoR2 WT-mRuby2 or N-CoR2 (ΔID2 CoRNR box)-mRuby2 (**panels 3** and **5**) were expressed in combination. This points towards co-localization and possible interaction of these two proteins. In cells expressing Clover-PPARγ1 in combination with N-CoR2 (ΔID1 exon)-mRuby2 or N-CoR2 (ΔID1 CoRNR box)-mRuby2, only a small number of weak spots on diffuse nuclei background were found for the Clover-PPARγ1 protein.

To quantify these observations, the PCC was used. As shown in **Figure 5C**, significant differences in PCC values are valid in HEK293T cells expressing Clover-PPARγ1 in combination with the different N-CoR2 constructs. Interestingly, the calculated PCC of Clover-PPARγ1 + N-CoR2 WT-mRuby2 (0.855 ± 0.086) was significantly higher compared to Clover-PPARγ1 + N-CoR2 (ΔID1 exon)-mRuby2 (0.532 ± 0.048) or Clover-PPARγ1 + N-CoR2 (ΔID1 CoRNR box)-mRuby2 (0.636 ± 0.048). In contrast, the PCC of Clover-PPARγ1 in combination with N-CoR2 WT-mRuby2 (0.855 ± 0.086) or N-CoR2 (ΔID2 CoRNR box)-mRuby2 (0.846 ± 0.048) remained almost unchanged. In comparison to these data, the background PCC values of cells expressing only the single fusion protein N-CoR2 WT-mRuby2 ID2/ID1 (0.326 ± 0.057) were significantly lower than the PCC data from simultaneously expressed protein couples (**Supplementary Figure 5**), indicating co-localization analyses of Clover-PPARγ1 with the single different N-CoR2 constructs is meaningful. To finally differentiate binding and/or co-localization, we used our flow cytometry-based-FRET assay system.

### Determination of protein-protein interactions of Clover-PPARγ1 and N-CoR2 WT-mRuby2 by flow cytometry-based FRET

**Figure 6:**
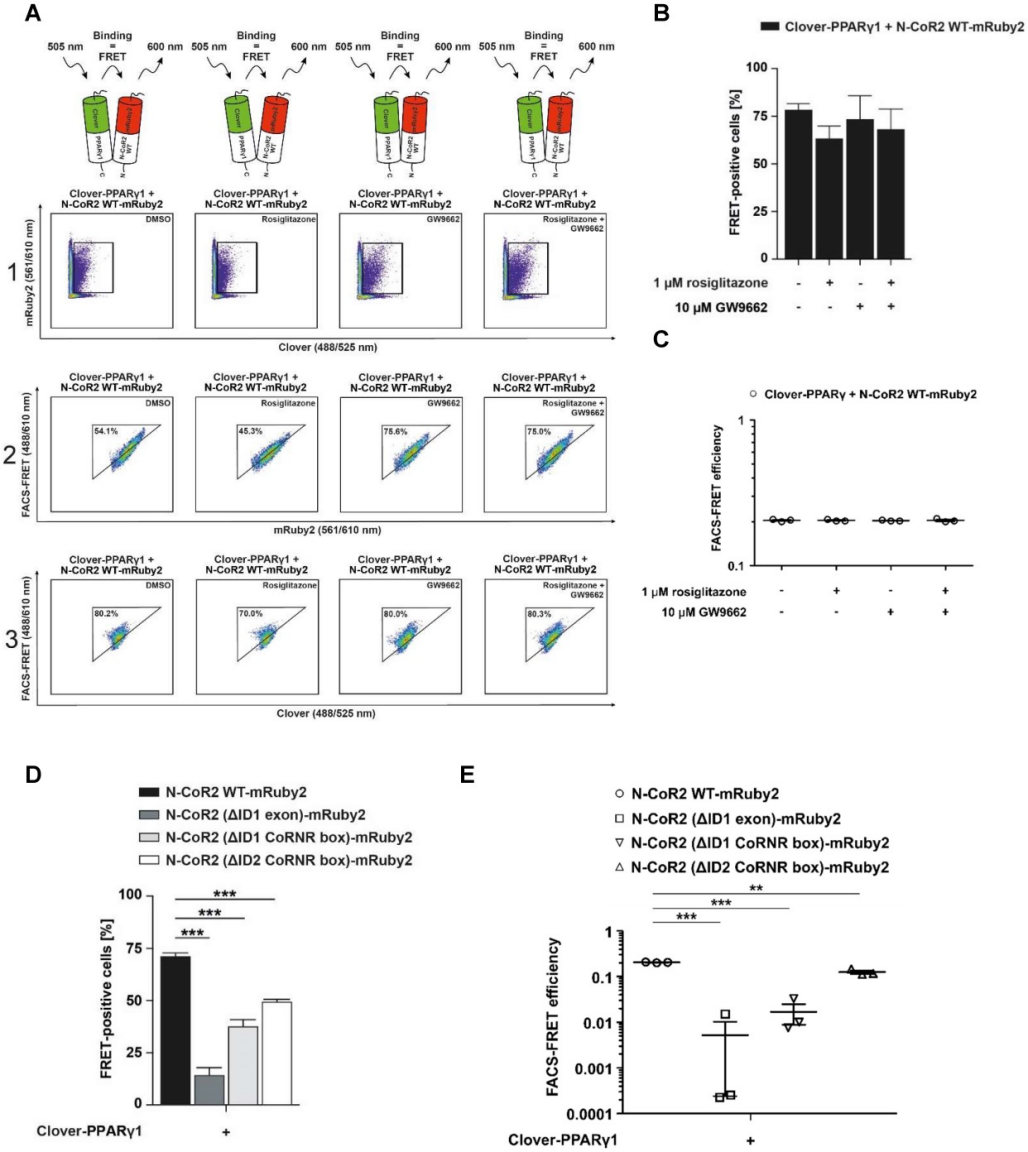
** Flow cytometry-based FRET-measurements to analyze protein-protein interactions between Clover-PPARγ1 and N-CoR2-mRuby2 constructs.** Representative primary flow cytometry-plots showing the quantity of FRET-positive living HEK293T Clover-PPARγ1 + N-CoR2 WT-mRuby2 cells are depicted (A). Images are representative of experiments which were performed three times. The cells were cultured for 24 h with DMSO, 1 µM of the PPARγ agonist, rosiglitazone, and 10 µM of the PPARγ antagonist, GW9662, alone or in combination. Panel 1 (1) represents the gating for Clover/mRuby2-double positive cells. Panel 2 (2) depicts cells positive for the first FRET gate of FRET 488/610nm vs. mRuby2 561/610 nm and panel 3 (3) shows cells with a FRET signal. Numbers in panels (2) and (3) represent total percentages of cells within the FRET gates. A comparison of the total percentages of FRET-positive living HEK293T Clover-PPARγ1 + N-CoR2 WT-mRuby2 cells is depicted in (B). The relative flow cytometry-based FRET efficiencies calculated as described in Materials and Methods are presented in (C). Comparison of the total percentages of FRET-positive living HEK293T Clover-PPARγ1 + N-CoR2 WT-mRuby2, Clover-PPARγ1 + N-CoR2 (ΔID1 exon)-mRuby2, Clover-PPARγ1 + N-CoR2 (ΔID1 CoRNR box)-mRuby2 and Clover-PPARγ1 + N-CoR2 (ΔID2 CoRNR box)-mRuby2 cells are depicted in (D). Flow cytometry-based efficiencies of Clover/mRuby2-double positive cells, calculated as described in Materials and Methods, are shown in (E). Values are means ± SD of three experiments. **P ≤ 0.01 and ***P ≤ 0.001.

Taking into consideration that N-CoR2 is a well-known PPARγ interaction partner 49, we investigated the effects of agonistic activation of PPARγ or its antagonism on this protein-protein interaction. For this, Clover-PPARγ1 and N-CoR2 WT-mRuby2 were stably expressed in HEK293T cells and treated for 24 h either with 1 µM rosiglitazone or 10 µM GW9662 or both added simultaneously. In **Figure 6A**, a representative flow cytometry-plot is shown. Transferring the previously described setup and gating strategy to the measurement of FRET by flow cytometry in living cells (**Figure 1F**) resulted in roughly the same number of FRET-positive cells expressing Clover-PPARγ1 and N-CoR2 WT-mRuby2 (**Figure 6B**), following solvent (**first dot blot**, **panel 3**), rosiglitazone (**second dot blot**, **panel 3**), GW9662 (**third dot blot**, **panel 3**) or combined rosiglitazone + GW9662 (**last dot blot**, **panel 3**) treatment.

In 2016, Schaufele determined the binding affinity of ligands based on the determination of the FRET intensity via the measurement of the mean relative fluorescence value 13. In relation to this important aspect, our FRET measurements revealed no differences in FRET intensity following treatment with an agonist or an antagonist, suggesting no altered affinity upon ligand binding. As shown in **Figure 6C**, flow cytometry-based FRET efficiencies were roughly similar after stimulation for 24 h with 10 µM of the PPARγ antagonist, GW9662, alone or in combination with 1 µM of the PPARγ agonist, rosiglitazone, or the PPARγ agonist rosiglitazone alone (**Figure 6C**). The gating strategy used is shown in** Supplementary Figure 6**.

Co-localization analyses suggest that specific N-CoR2 domains modulate PPARγ1 interaction (**Figure 5B** and **C**). We verified these results by flow cytometry-based FRET. As demonstrated previously, Clover-PPARγ1 and N-CoR2 WT-mRuby2 showed high FRET signals in the range of ~ 70%. Compared to N-CoR2 WT-mRuby2, the lack of ID1 exon (ΔAA 2310-2338), the ID1 CoRNR box (ΔAA 2329-2337) and the ID2 CoRNR box (ΔAA 2122-2130) significantly reduced the FRET signal, suggesting that these domains all contribute to NCoR2 binding (**Figure 6D**). Of note, the FRET results again exactly mirror the results from the co-localization analyses, but with much higher statistical confidence and background corrected. As a negative control, since there was no ligand binding in this latter experiment, we also analyzed whether these mutations alter the flow cytometry-based FRET efficiencies (**Figure 6E).** The gating strategy used is depicted in **Supplementary Figure 7**. As expected, the mutants showed less FRET with PPARγ1, in line with the number of FRET-positive cells, indicating a reduced overall number of total molecules that interact. Altogether, these data demonstrate that our assay is sensitive and reliable in measuring differences in overall protein-protein interactions (percentage of FRET-positive cells), as well as binding affinity between proteins in the presence or absence of ligands (flow cytometry-based FRET efficiency).

### Validation of the flow cytometry-based FRET system in murine J774A.1 macrophages

**Figure 7:**
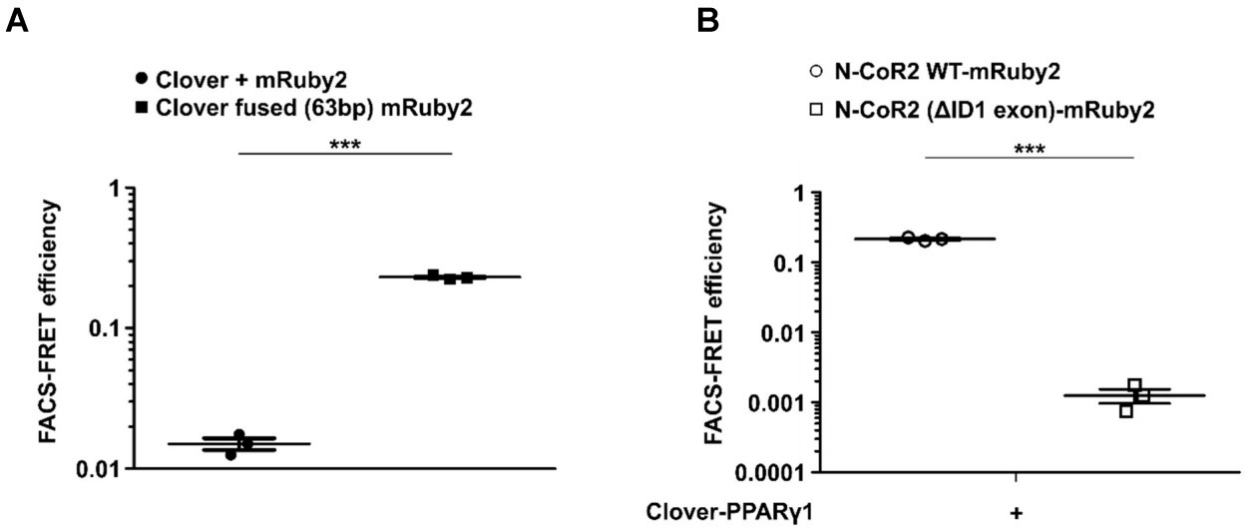
** Transfer of the flow cytometry-based FRET system to J774A.1 macrophages.** Flow cytometry-based FRET efficiency was determined in murine J774A.1 macrophages following transduction with **(A)** Clover + mRuby2 or Clover fused (63bp) mRuby2 and **(B)** Clover-PPARγ1 in combination with N-CoR2 WT-mRuby2 or N-CoR2 (ΔID exon)-mRuby2. Flow cytometry-based FRET efficiencies of Clover/mRuby2-double positive cells were calculated as described in Materials and Methods.

Based on our results obtained in HEK293T cells, we were interested to see, whether the flow cytometry-based FRET system can be used in more difficult to transfect cells as well. Therefore, we performed a final set of experiments using the murine macrophage cell line J774.A1. As shown in **Supplementary Figure 8**, gating for Clover/mRuby2-positive cells expressing Clover + mRuby2 and Clover fused (63bp) mRuby2 was performed. Fluorescence medians of Clover (488/525 nm), FRET (488/610 nm), and mRuby2 (561/610 nm) were used to calculate FRET efficiency, including medians of fluorescence negative J774A.1 cells as background control. Flow cytometry-based FRET efficiencies in J774A.1 cells (**Figure 7A**) were similar to the values in HEK293T cells (**Figure 1G**). Transduced J774A.1 cells expressing Clover-PPARγ1 and NCoR2 WT-mRuby2 or NCoR2 (ΔID1 exon)-mRuby2 were analyzed in analogy to HEK293T cells for protein-protein interaction of PPARγ1 and NCoR2. Following appropriate gating (**Supplementary Figure 9**), Clover/mRuby2-double-positive cells were analyzed for flow cytometry-based FRET. As shown in **Figure 7B**, similarly to HEK293T cells, the deletion of ID1 exon in NCoR2 prevented interaction with PPARγ1, which was visible with the NCoR2 WT-mRuby2 construct.

## Discussion

In 2010, Banning et al. described a new innovative flow cytometry-based FRET assay to detect protein-protein interactions in living cells 8. Compared to previous reports that combined flow cytometry and FRET 73, 74, Banning and colleagues designed an assay that allows quantitation and statistical analysis, eliminates cross talk artefacts and is easy to adapt and transferable to other applications 8. A unique advantage of such a flow cytometry-based FRET assay is that the FRET efficiency can be as easily quantified as the percentage of FRET-positive cells. Besides the many advantages offered by this assay, there are also some critical points that need to be taken into consideration. These include the fluorophores chosen, the steric orientation of the fluorophores, the expression and size of the fusion protein, the quantity of interacting proteins and finally, the distance between both interaction partners. One pivotal aspect of Banning´s flow cytometry-based FRET assay which could be improved, because it is a limiting factor, is the choice of the fluorophores. The FRET pair previously used, CFP/YFP, which are commonly used as donors and acceptors, respectively, have major limitations in their applicability. On the one hand, this is due to the pH-sensitivity of the YFP, which is tightly coupled to halide binding, and on the other, the multiple fluorescent states of CFP, its pH-dependence and low quantum yield 75, 76. To overcome most of the major problems, we sought to use a new, systematically developed FRET pair consisting of green and red fluorophores. FRET pairs using GFPs as donors and RFPs as acceptors are less commonly used, but exhibit fewer of the above mentioned disadvantages of CFP/YFP pairs 77-81. In the literature, it is reported that compared to the FRET pair CFP/YFP, the green/red fluorophore pairing of Clover/mRuby2 has an increased Förster distance and an improved dynamic range 14, 82, 83. In line with the findings of Lam and colleagues, using the “gold standard” method FLIM to verify FRET, we also confirmed that Clover/mRuby2 (**Figures 1D** and **E**) are an appropriate fluorophore pair for our cytometry-based FRET measurements. Our FLIM measurements show clearly the prolonged fluorescence lifetime of Clover compared to EGFP (**Supplementary Figure 10**). In addition, we were able to demonstrate a shift in the fluorescence lifetime of Clover resulting from changes in FRET activity. Such a shift, which is necessary for the FRET detection, could not be shown even for different AA linker lengths between EGFP and mRuby2 in our fluorophore fusion construct. As expected, we were able to easily transfer the adapted setup and gating strategy, using standard flow cytometry equipment, to measure FRET by flow cytometry in living cells with Clover/mRuby2. Thus, responses to these fluorophores either alone, in combination or fused were detectable as a high FRET of the positive control Clover fused (63bp) mRuby2 compared with the undetectable FRET from the negative control in HEK293T cells expressing Clover and mRuby2 simultaneously as distinct proteins (**Figure 1F** and** G** as well as** Supplementary Figure 1**).

The focus of current rational drug development is not only based on whether and where an active substance binds to cause changes in protein-protein interactions, but also on the affinity and the molecular features of the protein-protein interactions. In order to address these current questions, we modified and expanded the flow cytometry-based FRET system, developed by Banning and colleagues 8, with an additional variable. In addition to the findings in 2010 of Banning and colleagues 8, Schaufele determined binding affinity based on the analysis of the FRET intensity via measurement of the MFI value 13. To our knowledge, currently, no assay system has been described nor is available that combines the aspects of both systems. For this reason, we combined both assay systems for the investigation of binding intensity and affinity during the complex protein-protein interactions of PPARγ to its co-factors RXRα and NCoR2. This interaction plays a crucial role in the development of several obesity-related cancers and as a molecular and pharmacological target for autoimmune and inflammatory diseases 15-17.

PPARγ regulates gene expression upon heterodimerization with RXRα 35, 37. Thus, RXRα is the most important PPARγ co-factor. The significantly reduced FRET observed in HEK293T Clover-PPARγ1 + mRuby2-RXRα Δ414-462 compared to HEK293T Clover-PPARγ1 + mRuby2-RXRα cells (**Figures 3E, F,** and** G** as well as** Supplementary Figure 4**) showed clearly that such protein-protein interactions can be detected using our flow cytometry-based FRET assay system.

N-CoR2 is expressed ubiquitously and as an important PPARγ co-repressor it plays a crucial role in adipocyte differentiation and regulation of adipogenesis, insulin sensitivity, type 2 diabetes mellitus and PPARγ transcriptional activity 49, 50, 84. In recent years, a multitude of studies have described NHR - N-CoR2 binding and protein interactions 66, 67, 85, 86. Current reports show that NHRs generally bind more effectively to N‑CoR2 in the absence of ligands or in the presence of antagonists 49, 54. Thus, in addition to establishing the combined flow cytometry-based FRET system, we characterized the influence of specific PPARγ ligands on PPARγ1 - N-CoR2 binding and protein-protein interactions (**Figure 6A**). Interestingly, we found that in the absence (DMSO) or presence of the PPARγ antagonist, GW9662, no change in FRET-positive cells, in terms of total number of PPARγ1 - N-CoR2 binding and protein interactions, was observed (**Figure 6B**). The administration of the PPARγ agonist, rosiglitazone, also did not significantly alter the number of FRET-positive cells, indicating no change in PPARγ1 - N-CoR2 binding and protein interactions. This observation also confirms data in the current literature.

In the last few years, a variety of studies have shown that agonist binding to PPARγ results in a conformational change in the PPARγ receptor, leading to destabilization of co-repressor interactions and the loss of N-CoR2 binding and subsequent recruitment of co-activators 54, 55, 84. We propose that this explains our current findings. That the binding of the PPARγ agonist, rosiglitazone 54, 55, 84, 87 to the PPARγ ligand binding domain (LBD) results in a PPARγ conformational change, needs to be verified in further experiments. The consequence of this change and the loss of N-CoR2 binding was experimentally demonstrated, in our case, by a reduced number of FRET-positive cells. The binding of the PPARγ antagonist, GW9662 prevents this conformational change, stabilizes the protein interaction and thus maintains PPARγ1 - N-CoR2 binding and protein interactions, as reflected by a high number of FRET-positive cells. In addition, to verify the affinity of the PPARγ1 - N-CoR2 protein interactions and thereby, the effects of the PPARγ agonist, rosiglitazone, and antagonist, GW9662, in comparison to the absence of a ligand (DMSO), we introduced a new tool, determining the binding affinity based on the determination of the FRET intensity via the measurement of the MFI value 13. This modification revealed that neither antagonist nor agonist treatment altered PPARγ1 - N-CoR2 binding (**Figure 6C** and **Supplementary Figure 6**). Importantly, flow cytometry-based FRET efficiencies have to be calculated from raw flow cytometric data 10-12.

Physiologically relevant variations in the interactions between these two proteins exist with respect to the expressed protein isoforms. In the literature it has been reported that the N-CoR2 - NHR interaction, as well as its functional transcriptional co-repressor activity, is controlled by its protein structure. Crucial for these functions are the two C-terminal IDs (ID1 and ID2) of N-CoR2, that contain CoRNR box (NHR box) motifs which mediate the interaction between co-repressors and NHRs 13, 52, 54, 55, 84, 88. Furthermore, it has been reported that alternative splicing of single IDs or splicing among them has crucial consequences for their functions as an interaction partner of PPARγ 53-58. Because of the surprising result of the N-CoR2 amplification of HEK293T cells, in which we amplified the N-CoR2 (ΔID1 exon) variant, we further developed different N-CoR2 constructs to study the influence of selected AA deletions on PPARγ - N-CoR2 protein interactions.

With deletions in the N‑CoR2 interaction sequence, we detected significant differences in FRET-positive HEK293T cells. Compared to N-CoR2 WT-mRuby2, the lack of ID1 exon, CoRNR box ID1 (G…LEAIIRKALM), and CoRNR box ID2 (G…LAQHISEVI…K), which are essential for nuclear receptor binding to N-CoR2, significantly reduced the FRET signal. From these data, we assume that both interacting domains are important for the formation of a PPARγ/N-CoR2 complex. Furthermore, we observed that the deletion of only the ID1 motif itself (ID AA) decreased the binding capacity of the protein couple but not as efficiently as a complete lack of the ID1-containing exon of N-CoR2. Thus, it appears that some AA in the remaining ID1 exon are also important for stabilizing the protein-protein interaction or that the deletion of the exon mediates drastic changes in the quaternary structure of N-CoR2, which hampers the process of successful binding. As reported in the literature, our data indicate that the binding and the associated PPARγ - N-CoR2 protein interactions are strongly dependent on the N-CoR2 AA sequence, in which the interaction between this protein couples is mainly dictated by the ID1 CoRNR box motif. This aspect is also supported by the fluorescent microscopy analysis, which clearly showed a strong co-localization correlation only between PPARγ and the ID1 AA containing N-CoR2. However, where co-localization binding occurs, it appears to be with the same affinity as that during the flow cytometry-based FRET protein-protein interaction measurements, independently of the N-CoR2 AA modification.

Our cytometry-based FRET assay data, especially the outstanding role of ID1 observed during the interaction, were also supported and confirmed by studies of the mechanistic functionality of N-CoR2 WT-mRuby2 and N-CoR2 (ΔID1 exon)-mRuby2, using a PPARγ-dependent transactivation assay. Administration of the PPARγ agonist, rosiglitazone, led to an explicit PPARγ transactivation, whereas, in all cases, the administration of the PPARγ antagonist, GW9662, prevented the PPARγ transactivation, independently of the N‑CoR2 AA sequence modification. The analyses were done in HEK293T cells and therefore, control cells expressing only Clover-PPARγ without N-CoR2-mRuby2 inherit only the endogenous N-CoR2 with the missing ID1 exon, which results in nearly the same transactivation levels as in cells overexpressing N-CoR2 (ΔID1 exon)-mRuby2. In spite of the repressor nature of N-CoR2, we failed to detect a repression of transactivation in comparison to the control PPARγ alone. A possible explanation for this observation could be that the N-CoR2 constructs we utilized lack most of their regular N-Terminus (ΔRD1, SANT, ΔRD2), so that their AA sequence starts with RD3. As a result, N-CoR2 cannot interact to a full extent with other proteins of the normal repressor complex, like SIN3, the G-protein pathway suppressor (GPS2), transducin (beta)-like 1X-linked (TBL1)/transducin (beta)-like 1X-linked receptor 1 (TBLR1) and histone deacetylase 3 (HDAC3) and therefore, the repression of transcription is lost 89, 90. Nevertheless, with this assay, we could show that the ID1 domain of N-CoR2 enhances the functional outcome of the PPARγ - N-CoR2 interaction, by leading to a strong transactivation rate in rosiglitazone treated cells. This conclusion is well supported by the significant FRET reduction of Clover-PPARγ1 + N-CoR2 (ΔID1 exon)-mRuby2 and also by the absent co-localization of this protein couple in living HEK293T cells. Further detailed studies are needed to elucidate mechanistic details.

We finally proved that our flow cytometry-based FRET system can also be used in cells which can barely be transfected/transduced compared to HEK293T cells. Thus, in murine J774A.1 macrophages, the flow cytometry-based FRET assay revealed similar results compared to HEK293T cells, although the number of double positive cells was significantly lower. These data support the notion that our system is also suitable to determine protein-protein interactions in cells which are more difficult to handle.

NHR such as PPARγ1 are directly involved in many disease pathways. Most of them have been the subject of screening assays to detect protein-protein interactions and to identify compounds for the characterization of co-factor recruitment and selective modulators. For practical reasons, such efforts at drug development have mainly relied on cell-free receptor homo- or heterodimers on DNA 91. Moreover, the major readout for compound screening assays has been co-regulator recruitment. These studies have been guided by the assumption that different NHR ligands can impart their gene-selective actions strictly through their differential impact on co-regulator affinities. Given that co-regulator availability differs in cell types, the altered affinities are thought to give rise to selective modulator properties in these ligands.

## Conclusions

In this report, we clearly show that our innovative, systematically improved, cytometry-based FRET assay provides detailed, reliable, reproducible data and allows the molecular identification and characterization of direct protein-protein interactions of the human nuclear factor PPARγ1 with its co-factors in living cells as well as permitting identification of novel protein interaction partners. The setting opens the possibility of transfer to other NHR to identify their binding characteristics to co-activators and co-repressors also in an HTS approach. Thus, this assay is expected to contribute to the identification of a variety of novel therapeutic targets for treatment of human diseases.

## Supplementary Material

Supplementary figures and tables.

## Abbreviations

A: adenine
AA: amino acid
ADA2: adenosine deaminase 2
CBP: cyclic adenosine monophosphate response element binding protein
CCD: charge-coupled device
CFP: cyan fluorescent protein
CLSM: confocal laser scanning microscope
CO_2_: carbon dioxide
DMEM: Dulbecco's Modified Eagle's Medium
CoRNR: co-repressor nuclear receptor
DMSO: dimethyl sulfoxide
DNA: deoxyribonucleic acid
FCS: fetal calf serum
FLIM: fluorescence lifetime imaging microscopy
FRET: Förster´s resonance energy transfer
FSC: forward scatter
GFP: green fluorescent proteins
GPS2: G-protein pathway suppressor
GW9662: 2-chloro-5-nitrobenzanilide
HDAC3: histone deacetylase 3
HEK293T: human embryonic kidney 293 cells stably expressing the large T antigen of simian vacuolating virus 40
HTS: high-throughput-screening
ID: interaction domain
LBD: ligand binding domain
MFI: median fluorescence intensity
N-CoR2: nuclear receptor co-repressor 2
NHR: nuclear hormone receptor
OFP: orange fluorescent protein
PBS: phosphate-buffered saline
PCC: Pearson´s correlation coefficient
PCR: polymerase chain reaction
PE: phycoerythrin
PFA: paraformaldehyde
PPARγ1: peroxisome proliferator-activated receptor gamma 1
PPREs: PPARγ response elements
RD: regulation domain
RFP: red fluorescent protein
RPMI: Roswell Park Memorial Institute
RXRα: retinoid X receptor alpha
SANT: SWI3 - ADA2 - N-CoR - TFIIIB
SD: standard deviation
SDS: sodium dodecyl sulfate
SMRT: silencing mediator of retinoic acid and thyroid hormone receptor
SRC1: steroid receptor co-activator 1
SSC: side scatter
T: thymine
TBL1: transducin (beta)-like 1X-linked
TBLR1: transducin (beta)-like 1X-linked receptor 1
TCSPC: time-correlated single photon counting
TFIIIB: transcription factor IIIB
TR**: time-resolved
YFP: yellow fluorescent protein
