# Supplementary material for: Flow cytometry-based FRET identifies binding intensities in PPARγ1 protein-protein interactions in living cells
Source: Theranostics. 2019 Jul 28;9(19):5444–63. doi: 10.7150/thno.29367 (PMC6735382; doi:10.7150/thno.29367)
Supplement: Supplementary file 1 — Supplementary figures and tables. [file thnov09p5444s1.pdf]

Supplementary Figures 1-10

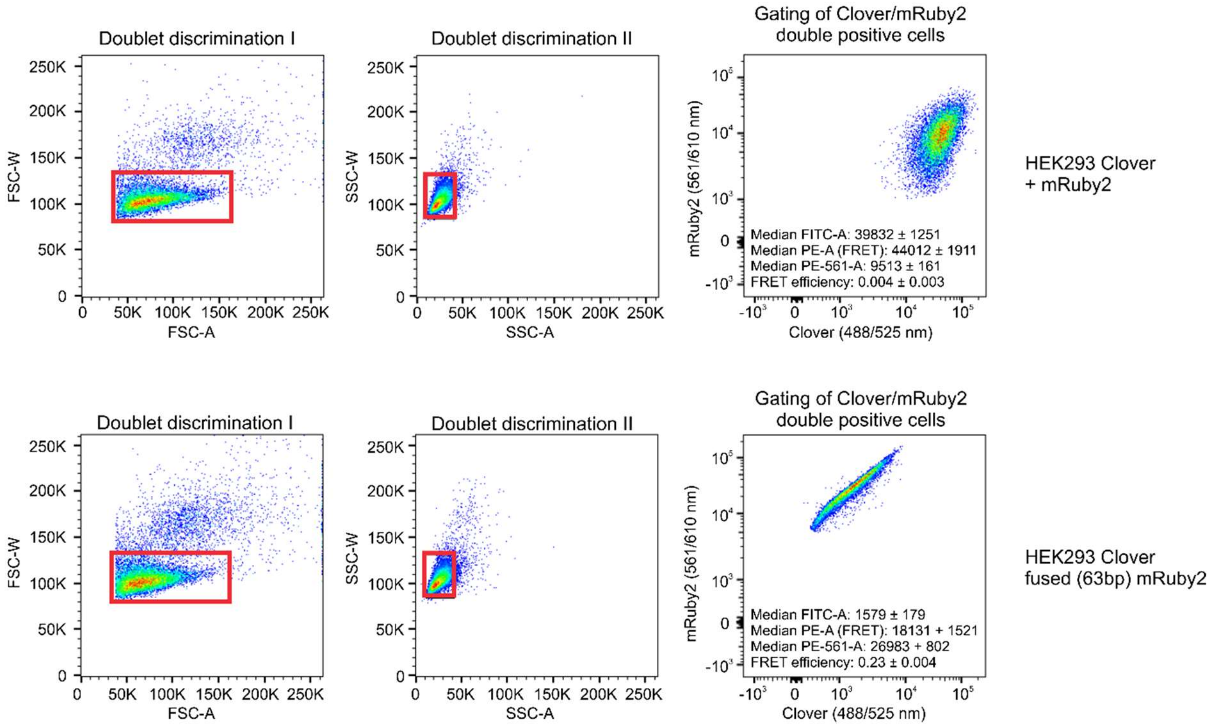

Supplementary Figure 1. Determination of FACS-FRET efficiencies. HEK293T cells were stably transduced with the Clover + mRuby2 as well as the Clover fused (63bp) mRuby2 and analyzed using a flow cytometer. Doublet discrimination was done by gating for FSC-W vs. FSC-A followed by SSC-W vs. SSC as shown in the first two panels. Single cells were further analyzed to be double positive for Clover and mRuby2 (panel 3). FRET efficiencies, depicted as inlets in the third panel, were calculated based on fluorescence medians of Clover (488/525 nm), FRET (488/610 nm), and mRuby2 (561/610 nm), also shown in the third panel, using the FRET calculator protocol as described in Materials and Methods. Flow cytometry-plots are representative for experiments which were performed at least three times.

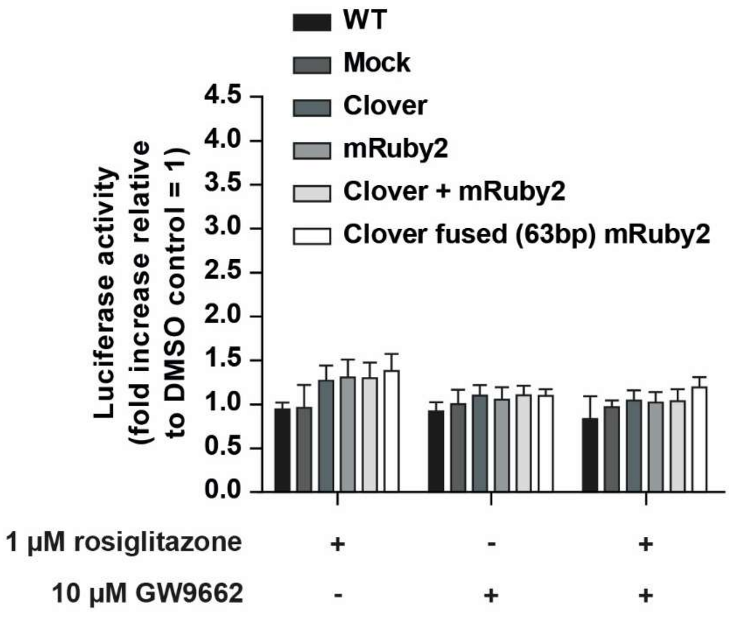

Supplementary Figure 2. Mechanistic functionality of the FRET pair Clover/mRuby2. A PPARγ-dependent transactivation assay was used to verify the mechanistic functionality of HEK293T cells expressing Clover/mRuby2 alone or in combination, Clover fused (63bp) mRuby2. Untransfected WT and mock transfected cells were used as controls. HEK293T cells expressing protein(s) as indicated were stimulated for 24 h with 1 µM rosiglitazone alone or in combination with 10 µM GW9662. Values from PPARγ-dependent transactivation experiments are means ± SD of three to seven individually experiments. Each PPARγ-dependent transactivation assay experiment was performed in quadruple.

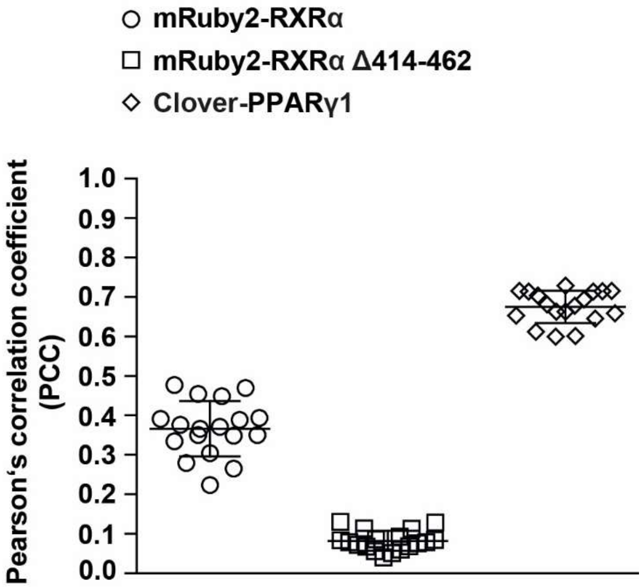

Supplementary Figure 3. The fluorescence and cellular localization of Clover-PPARγ1, mRuby2-RXRα and mRuby2-RXRα Δ414-462. The PCC was used for the correlation quantification of the subcellular co-localization of HEK293T cells stably expressing Clover-PPARγ1, mRuby2-RXRα or mRuby2-RXRα Δ414-462 as single fusion proteins. R-Values are means ± SD of 18 individual cells.

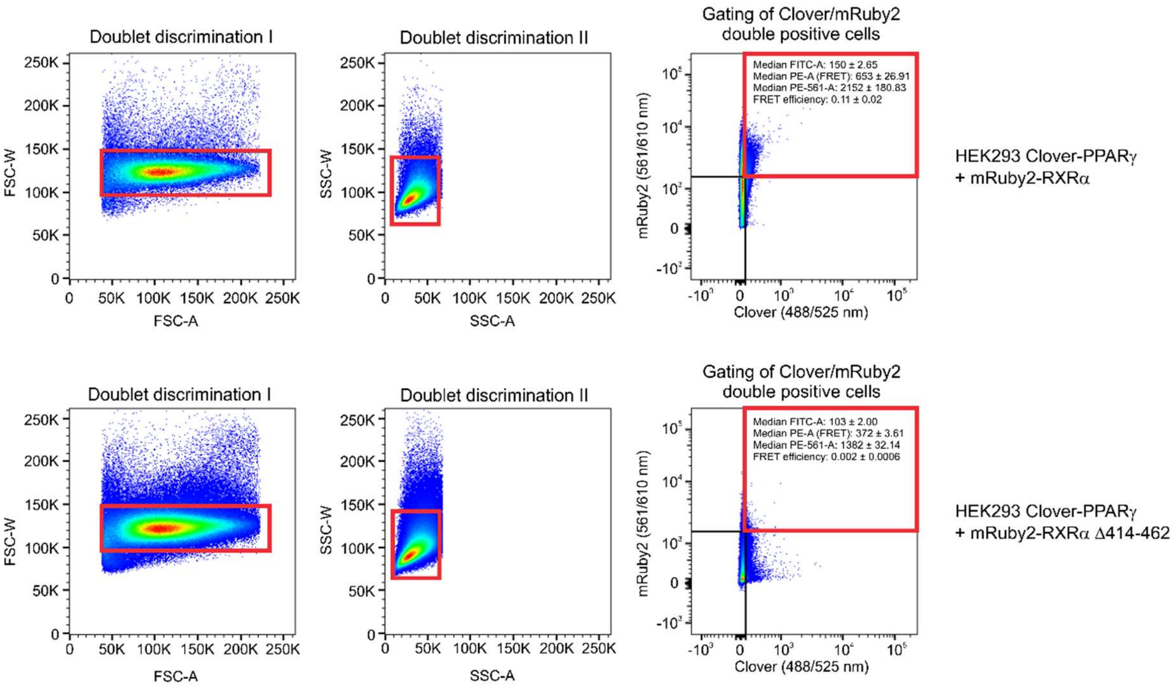

Supplementary Figure 4. Determination of FRET efficiencies of PPARγ binding to RXRα. HEK293T cells were stably transduced with the Clover-PPARγ and mRuby2-RXRα constructs and analyzed using a flow cytometer. Doublet discrimination was done by gating for FSC-W vs. FSC-A followed by SSC-W vs. SSC as shown in the first two panels. Single cells were further analyzed to be double positive for Clover and mRuby2 (panel 3, upper right quadrant). FRET efficiencies, depicted as inlets in this upper right quadrant, were calculated based on fluorescence medians of Clover (488/525 nm), FRET (488/610 nm), and mRuby2 (561/610 nm), of the upper right quadrant also shown in the third panel, using the FRET calculator protocol as described in Materials and Methods. Flow cytometry-plots are representative for experiments which were performed at least three times.

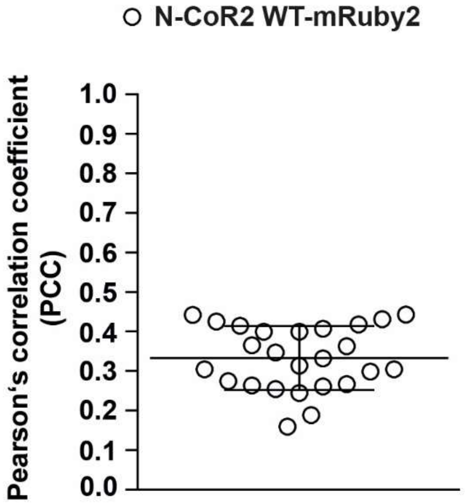

Supplementary Figure 5. The fluorescence and cellular localization of N-CoR2 WT-mRuby2. The PCC was used for the correlation quantification of the subcellular co-localization of HEK293T cells stably expressing N-CoR2 WT-mRuby2 as a single fusion protein. R-Values are means ± SD of 25individual cells.

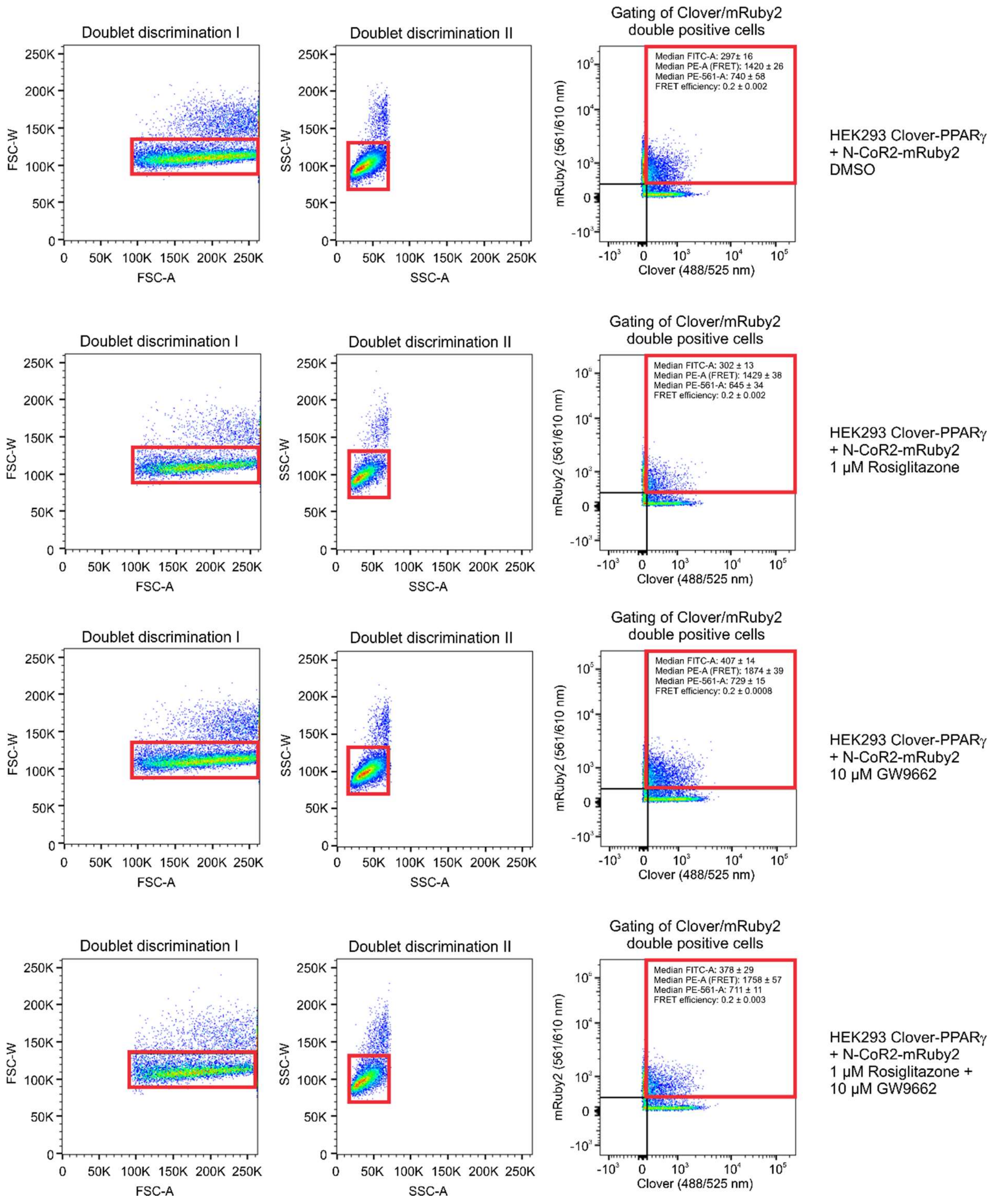

Supplementary Figure 6. Determination of FACS-FRET efficiencies of Clover-PPARγ1 and N-CoR2 WT-mRuby2. HEK293T cells were stably transduced with the Clover-PPARγ and N-CoR2 WT-mRuby2 constructs, treated as indicated for 24 h and analyzed using a flow cytometer. Doublet discrimination was done by gating for FSC-W vs. FSC-A followed by SSC-W vs. SSC as shown in the first two panels. Single cells were further analyzed to be double positive for Clover and mRuby2 (panel 3, upper right quadrant). FACS-FRET efficiencies, depicted as inlets in this upper right quadrant, were calculated based on fluorescence medians of Clover (488/525 nm), FRET (488/610 nm), and mRuby2 (561/610 nm), of the upper right quadrant (also shown there), using the FRET calculator protocol as

described in Materials and Methods. Flow cytometry-plots are representative for experiments which were performed at least three times.

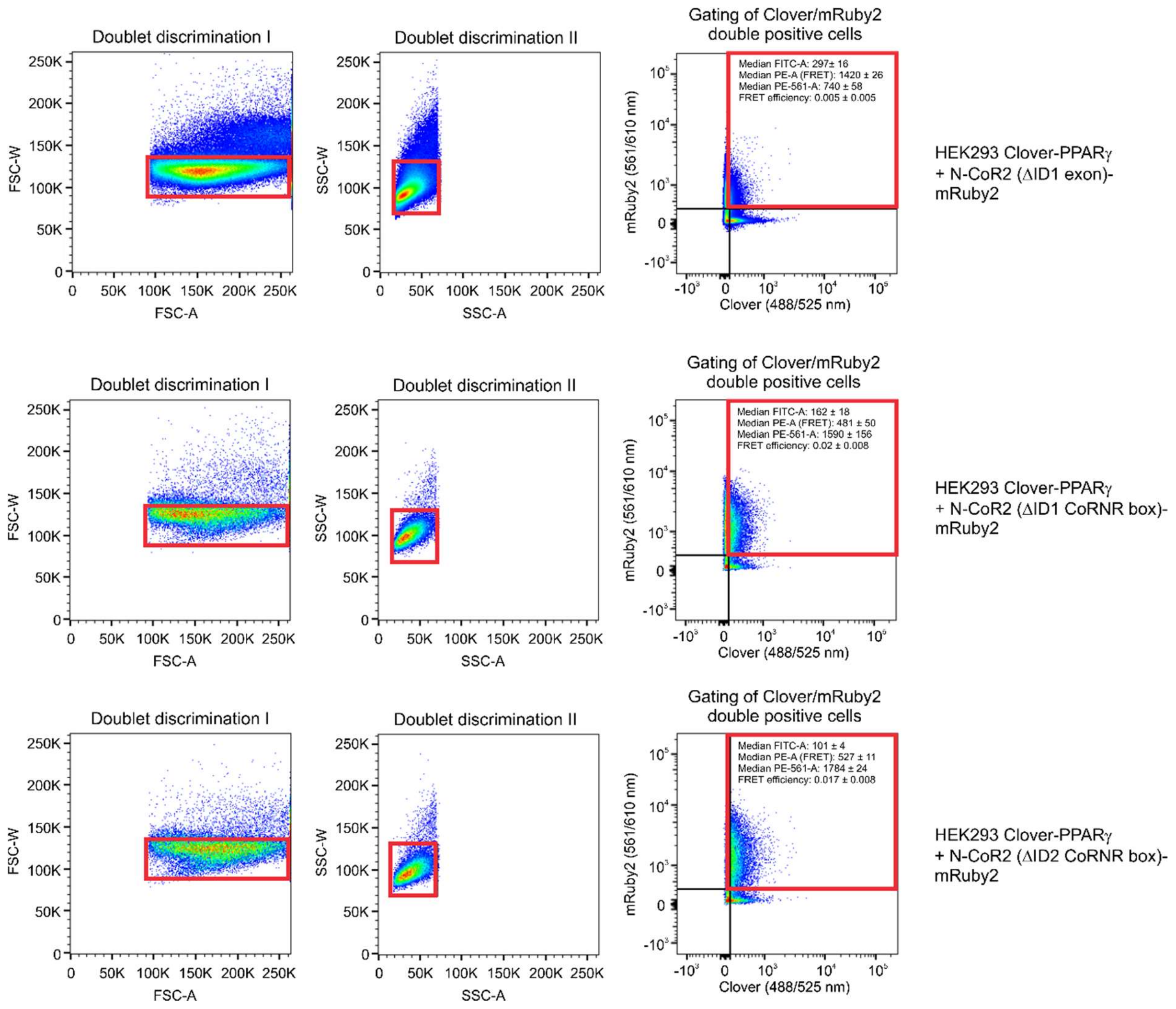

Supplementary Figure 7. Determination of FACS-FRET efficiencies of Clover-PPARγ1 and N-CoR2-mRuby2 constructs. HEK293T cells were stably transduced with the Clover-PPARγ and N-CoR2-mRuby2 constructs as indicated and analyzed using a flow cytometer. Doublet discrimination was done by gating for FSC-W vs. FSC-A followed by SSC-W vs. SSC as shown in the first two panels. Single cells were further analyzed to be double positive for Clover and mRuby2 (panel 3, upper right quadrant). FACS-FRET efficiencies, depicted as inlets in this upper right quadrant, were calculated based on fluorescence medians of Clover (488/525 nm), FRET (488/610 nm), and mRuby2 (561/610 nm), of the upper right quadrant (also shown there), using the FRET calculator protocol as described in Materials and Methods. Flow cytometry-plots are representative for experiments which were performed at least three times.

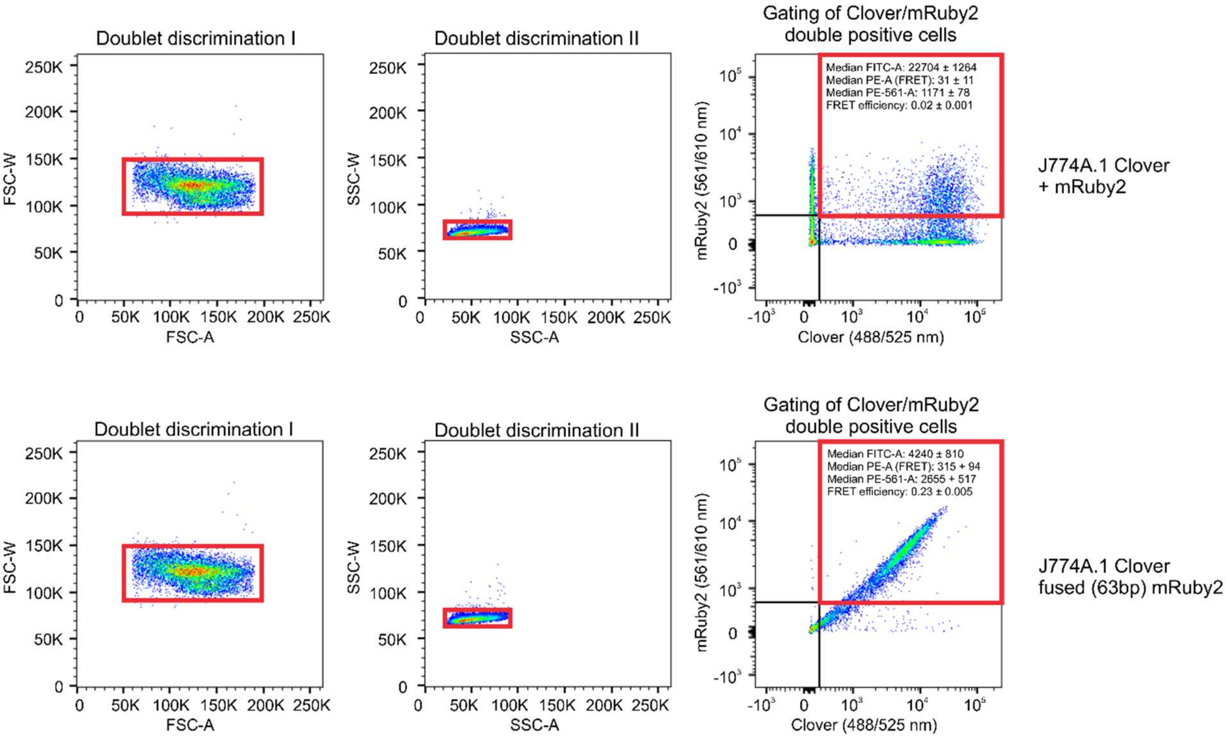

Supplementary Figure 8. Determination of FACS-FRET efficiencies in murine J774A.1 macrophages. J774A.1 cells were transduced with the Clover + mRuby2 as well as the Clover fused (63bp) mRuby2 and analyzed using a flow cytometer. Doublet discrimination was done by gating for FSC-W vs. FSC-A followed by SSC-W vs. SSC as shown in the first two panels. Single cells were further analyzed to be double positive for Clover and mRuby2 (panel 3). FRET efficiencies, depicted as inlets in the third panel, were calculated based on fluorescence medians of Clover (488/525 nm), FRET (488/610 nm), and mRuby2 (561/610 nm), also shown in the third panel, using the FRET calculator protocol as described in Materials and Methods. Flow cytometry-plots are representative for experiments which were performed at least three times.

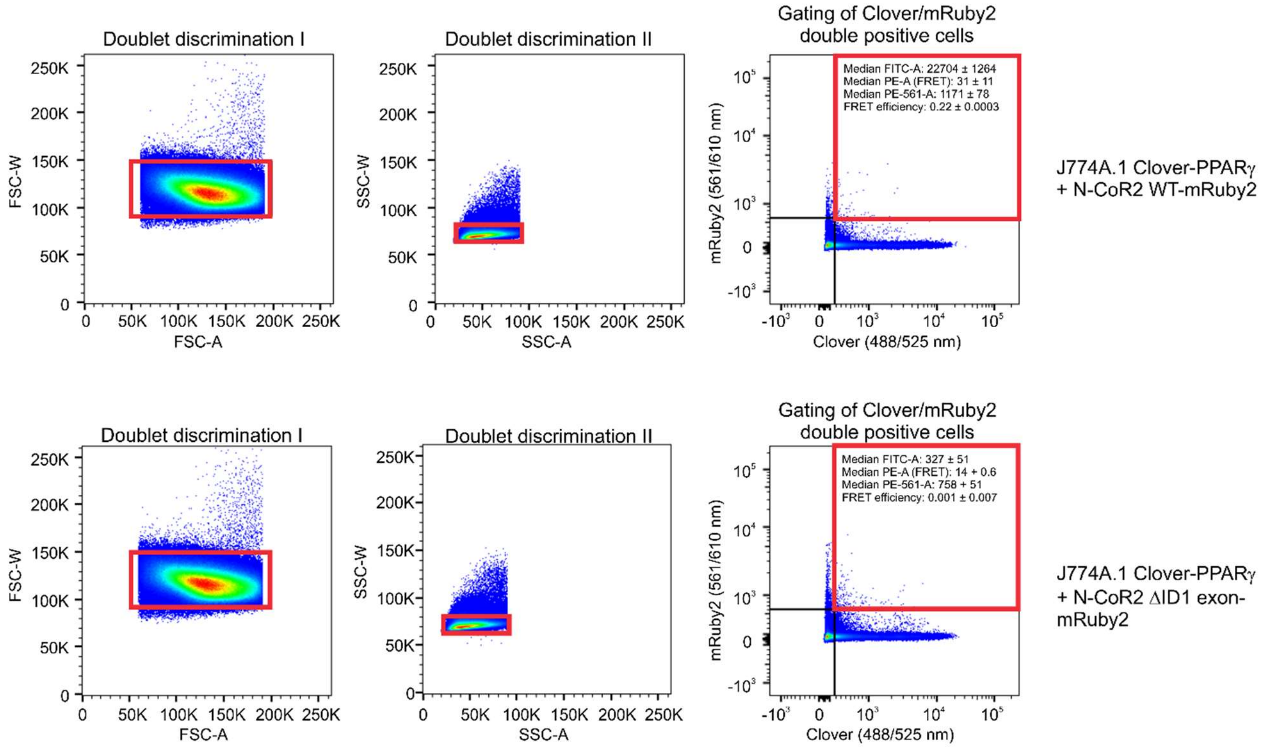

Supplementary Figure 9. Determination of FACS-FRET efficiencies of Clover-PPARγ1 and N-CoR2-mRuby2 constructs. J774A.1 cells were transduced with the Clover-PPARγ and N-CoR2 WT-mRuby2 and N-CoR2 ΔID1 exon-mRuby2 constructs as indicated and analyzed using a flow cytometer. Doublet discrimination was done by gating for FSC-W vs. FSC-A followed by SSC-W vs. SSC as shown in the first two panels. Single cells were further analyzed to be double positive for Clover and mRuby2 (panel 3, upper right quadrant). FACS-FRET efficiencies, depicted as inlets in this upper right quadrant, were calculated based on fluorescence medians of Clover (488/525 nm), FRET (488/610 nm), and mRuby2 (561/610 nm), of the upper right quadrant (also shown there), using the FRET calculator protocol as described in Materials and Methods. Flow cytometry-plots are representative for experiments which were performed at least three times.

A

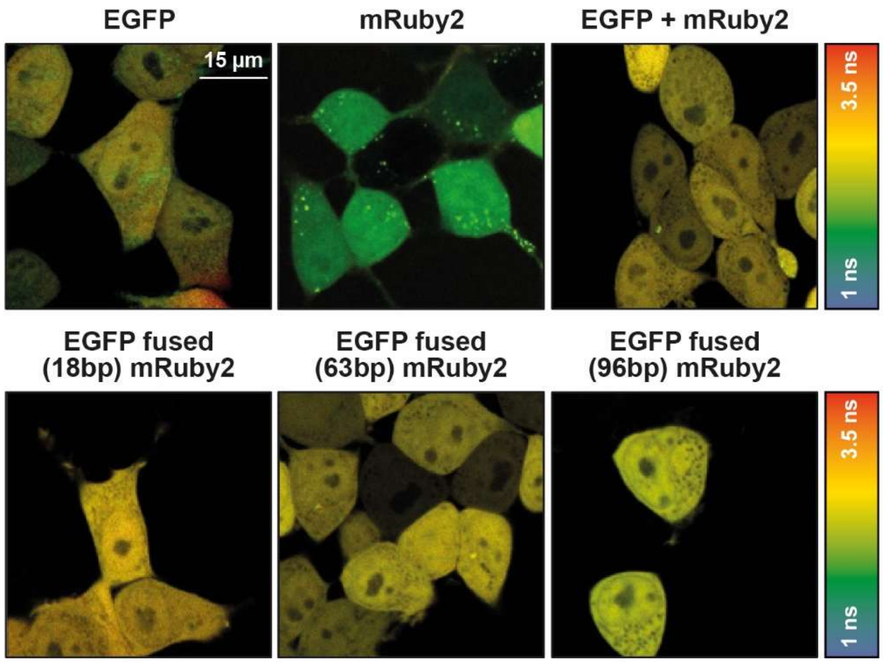

B

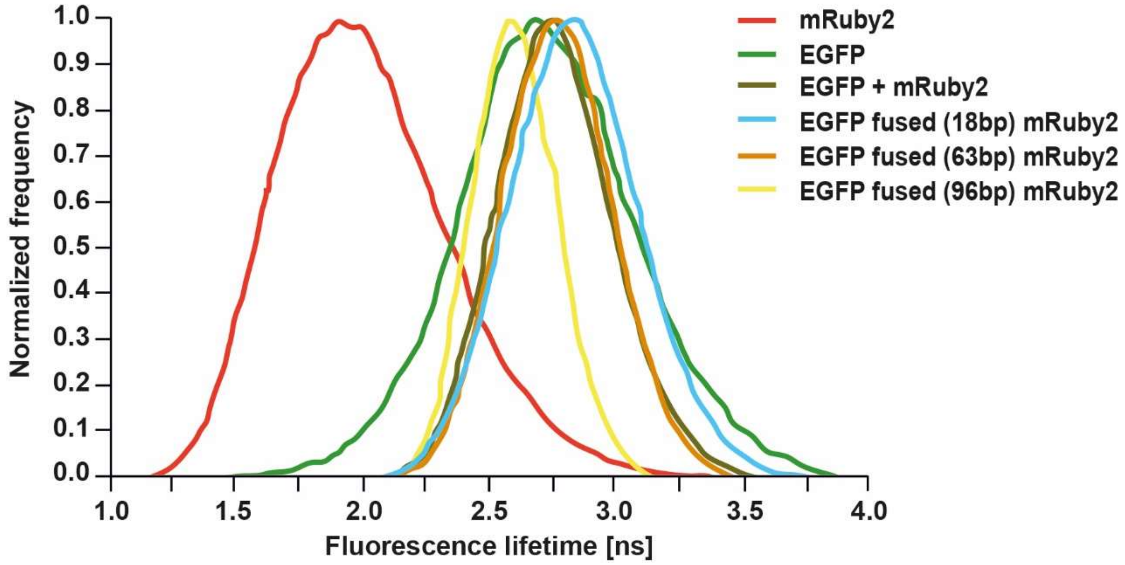

Supplementary Figure 10. Verification of EGFP, mRuby2 and EGFP fused (18bp)/(63bp)/(96bp) mRuby2 for flow cytometry FRET measurements. FLIM images **(A)** of HEK293T cells with the controls EGFP, mRuby2, EGFP + mRuby2 and EGFP fused (18bp)/(63bp)/(96bp) mRuby2. The histogram **(B)** shows the normalized frequency of fluorescence lifetimes in the images. Images and the histogram are representative for experiments which were performed at least three times.
